# Transient kinetics reveal the mechanism of competitive inhibition of the neutral amino acid transporter ASCT2

**DOI:** 10.1016/j.jbc.2024.107382

**Published:** 2024-05-17

**Authors:** Yang Dong, Jiali Wang, Christof Grewer

**Affiliations:** Department of Chemistry, Binghamton University, Binghamton, New York, USA

**Keywords:** neutral amino acid transporter, ASCT2, electrophysiology, kinetics, rapid solution exchange, membrane protein, inhibition mechanism

## Abstract

ASCT2 (alanine serine cysteine transporter 2), a member of the solute carrier 1 family, mediates Na^+^-dependent exchange of small neutral amino acids across cell membranes. ASCT2 was shown to be highly expressed in tumor cells, making it a promising target for anticancer therapies. In this study, we explored the binding mechanism of the high-affinity competitive inhibitor *L*-*cis* hydroxyproline biphenyl ester (*Lc*-BPE) with ASCT2, using electrophysiological and rapid kinetic methods. Our investigations reveal that *Lc*-BPE binding requires one or two Na^+^ ions initially bound to the *apo*-transporter with high affinity, with Na1 site occupancy being more critical for inhibitor binding. In contrast to the amino acid substrate bound form, the final, third Na^+^ ion cannot bind, due to distortion of its binding site (Na2), thus preventing the formation of a translocation-competent complex. Based on the rapid kinetic analysis, the application of *Lc*-BPE generated outward transient currents, indicating that despite its net neutral nature, the binding of *Lc*-BPE in ASCT2 is weakly electrogenic, most likely because of asymmetric charge distribution within the amino acid moiety of the inhibitor. The preincubation with *Lc*-BPE also led to a decrease of the turnover rate of substrate exchange and a delay in the activation of substrate-induced anion current, indicating relatively slow *Lc*-BPE dissociation kinetics. Overall, our results provide new insight into the mechanism of binding of a prototypical competitive inhibitor to the ASCT transporters.

The solute carrier 1 (SLC1) family is a group of membrane transport proteins including five high-affinity glutamate transporters (excitatory amino acid transporters, EAATs) and two neutral amino acid transporters (alanine serine cysteine transporters, ASCTs) (1, 2, 3). These SLC1 transporters mediate the uptake and efflux of amino acids by cells. ASCT2 (alanine serine cysteine transporter 2) was previously shown to function as a neutral amino acid exchanger (2). ASCT2 plays a crucial role in facilitating the uptake of glutamine, which serves as a vital source of energy and nitrogen for the proliferation of rapidly growing tumor cells (4, 5). In many tumor cell types, ASCT2 is highly upregulated, such as in hepatocellular carcinoma, colorectal cancer, and breast cancer (6, 7, 8). For this reason, ASCT2 is emerging as a promising anticancer drug target. Therefore, a series of competitive and noncompetitive inhibitors targeting ASCT2 have been developed.

SLC1 family members are Na^+^-dependent transporters, however, the ion coupling stoichiometries differ between the neutral and acidic amino acid transporters in the family. In the EAATs, glutamate is cotransported with three Na^+^ ions and one proton and one K^+^ ion is counter-transported (9, 10, 11, 12), while in ASCTs, the uptake of one neutral amino acid is in exchange of countertransported amino acid, modulated by the binding of two or three Na^+^ ions to the transporter (3, 13, 14). ASCTs and EAATs share a high similarity of the amino acid sequence in the transmembrane domains. The substrate binding mechanism of EAATs has been thoroughly studied. In the substrate-free state, HP2 (hairpin loop 2) is closed; subsequently, two Na^+^ ions bind to the transporter. This is followed by remodeling of the substrate binding site, facilitating the opening of HP2 for substrate binding. Once the substrate is bound, HP2 closes again. Finally, the binding of the third Na^+^ occurs (15, 16, 17, 18, 19). The substrate/Na^+^ binding mechanism for ASCTs, on the other hand, has not been comprehensively characterized.

Currently, several competitive inhibitors have been identified, based on structural similarity with competitive EAAT inhibitors (20). A variety of different amino acid-based scaffolds were explored, for example, benzylproline derivatives, p-nitrophenyl glutamyl derivatives, and serine-based derivatives (21, 22, 23). Among these ASCT2 inhibitors, *L*-*cis* hydroxyproline biphenyl ester (*Lc*-BPE) is one of the most potent, demonstrating an apparent *K*_i_ value of 0.74 μM in rat ASCT2 (rASCT2) and 0.86 μM in human ASCT2 (hASCT2) (24). *Lc*-BPE was developed in our laboratory in 2021, when we combined computational modeling and experimental approaches to identify a distinct subpocket within the substrate binding site. The cryo-EM structure of the hASCT2-*Lc*-BPE complex captures a pharmacologically relevant outward-open conformation (24). The structure suggests that *Lc*-BPE is a competitive inhibitor binding to the substrate binding site from the extracellular side. In addition, several other ASCT2 structures were published, including inward- and outward-facing conformations with or without substrate bound (25, 26, 27). One of these structures suggests a one-gate elevator mechanism, in which HP2 functions as the only gate, which controls access to the binding site in both inward- and outward-facing conformations (27).

In this study, we applied electrophysiological techniques to investigate the kinetic properties of the steady-state and presteady-state currents associated with ASCT2 function. *Lc*-BPE is utilized as a tool to characterize the binding mechanism and electrostatics of sodium, substrate, and competitive inhibitors in ASCT2. The goals were to identify the functional similarities/differences between substrate/competitive inhibitor binding to ASCT2 and their implications for the structural mechanism of Na^+^ and ligand interaction with the transporter. Our results indicate a transport mechanism wherein one or two Na^+^ ions initially bind to the unoccupied transporter (likely Na1 and Na3 sites), subsequently followed by the binding of either an amino acid or a competitive inhibitor. For amino acid substrates, an additional Na^+^ binding results in the formation of a complex that is competent for translocation. However, for competitive inhibitors, this subsequent Na^+^ binding step cannot take place. This is likely due to competitive inhibitors preventing the closure of HP2. We quantitatively explain the outward charge movement created by inhibitor binding, caused by movement of the zwitterionic amino acid moiety into the binding site, with the negatively charged carboxy group more deeply penetrating the transmembrane electric field. Together, these findings shed additional light on the mechanistic details of the inhibitor/transporter interaction, possibly facilitating the development of more potent competitive inhibitors in the future.

## Results

### Binding of *Lc*-BPE is associated with a partially occupied Na^+^ binding state

**Figure 1:**
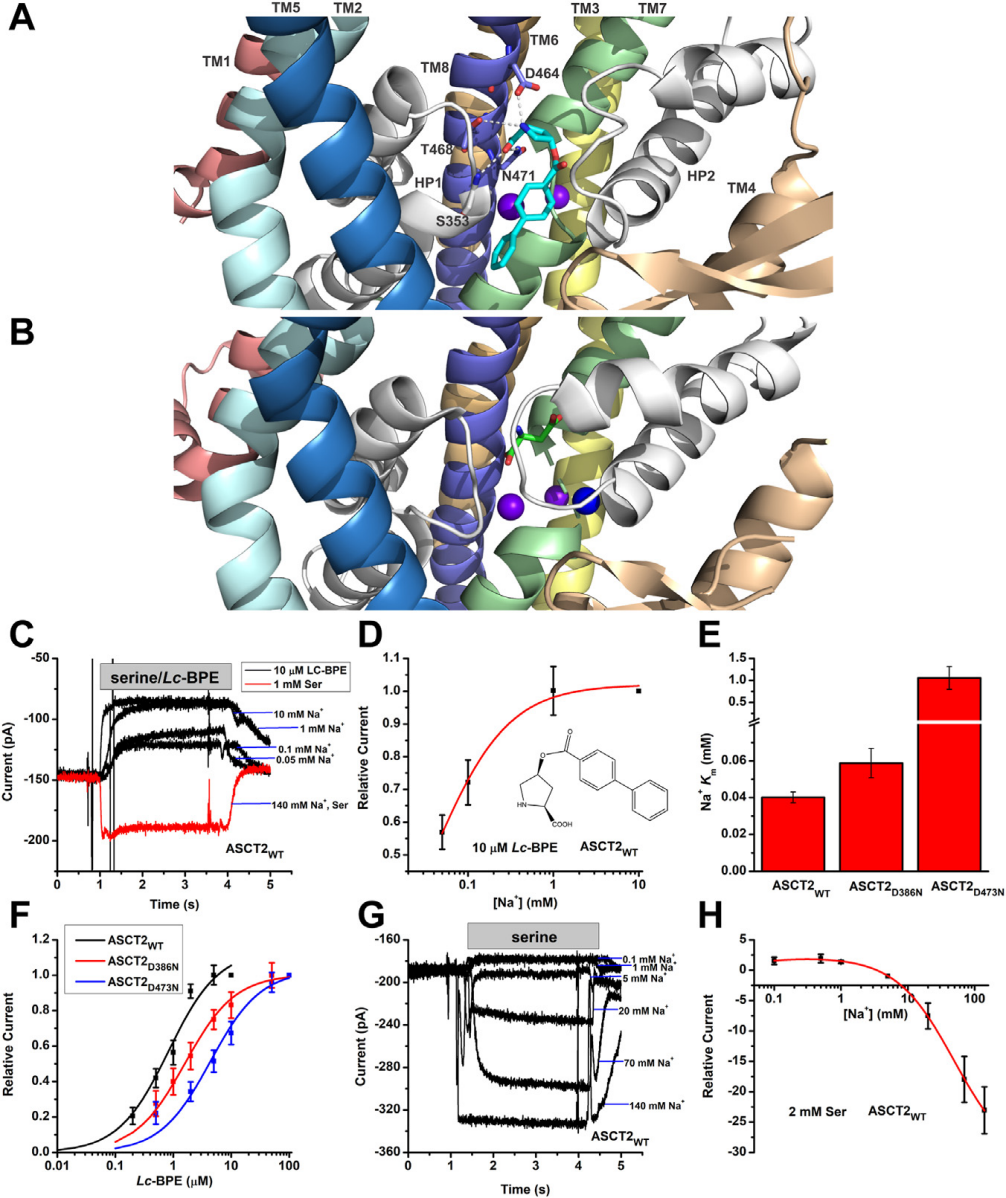
**The [Na**^**+**^**] dependence of *Lc*-BPE-induced current is compatible with a single-Na**^**+**^**site model.***A*, *Lc*-BPE (*cyan sticks*) binding site of ASCT2 (7BCS, “ligand-down” orientation, HP2 *open*). HP1 and HP2 are shown in *white*. Predicted sodium ions in Na1/Na3 sites are shown as *purple spheres*. *B*, substrate binding site of EAAT1 (7AWM, HP2 *closed*). L-Asp is shown as *green sticks* and sodium ions in Na1/Na3 sites are shown as *purple spheres*, in Na2 site is shown as a *blue sphere*. *C*, typical *Lc*-BPE-induced ASCT2 anion current (*black*) at increasing Na^+^ concentrations. The extracellular solution contained 0.05, 0.1, 1, or 10 mM NaMes (sodium methanesulfonate) and 10 μM *Lc*-BPE, the internal solution contained 130 mM NaSCN (sodium thiocyanate)/10 mM serine. The current illustrated by the *red trace* was induced by 2 mM serine in the presence of 140 mM of extracellular NaMes as a control. The application time for *Lc*-BPE or serine is indicated by the *gray bar*. *D*, [Na^+^] dose-response curve at 10 μM *Lc*-BPE for ASCT2_WT_. The *red line* represents the best nonlinear curve fit to a Michaelis–Menten-like equation. The apparent affinity for Na^+^ was calculated as *K*_m_ = 0.04 ± 0.003 mM. The inset shows the structure of *Lc*-BPE. *E*, apparent Na^+^*K*_m_ values for ASCT2_WT_, ASCT2_D386N_, and ASCT2_D473N_ in the presence of 100 μM *Lc*-BPE. *F*, [*Lc*-BPE] dose-response curves for ASCT2_WT_, ASCT2_D386N_, and ASCT2_D473N_ in the presence of 140 mM Na^+^, with apparent *K*_m_ value of 0.83 ± 0.2 μM for ASCT2_WT_, 1.6 ± 0.1 μM for ASCT2_D386N_, 4.4 ± 0.8 μM for ASCT2_D473N_. *G*, typical serine-induced ASCT2 anion current at increasing Na^+^ concentrations. *H*, [Na^+^] dose-response curve at 2 mM serine for ASCT2_WT_. Anion currents were fitted using a two site Michaelis–Menten like equation, $I=I_{1}\frac{\left[{Na}^{+}\right]}{\left(K_{m1}+\left[{Na}^{+}\right]\right)}+I_{2}\frac{\left[{Na}^{+}\right]}{\left(K_{m2}+\left[{Na}^{+}\right]\right)}$. The apparent affinities for Na^+^ were calculated as *K*_m__1_ = 0.04 ± 0.02 mM and *K*_m__2_ = 49 ± 2 mM. The membrane potential was 0 mV in all experiments. ASCT, alanine serine cysteine transporter; EAAT, excitatory amino acid transporter; HP2, hairpin loop 2; *Lc*-BPE, *L*-*cis* hydroxyproline biphenyl ester.

*Lc*-BPE has been previously reported to be a potent competitive, stereo-selective inhibitor of ASCT2. The cryo-EM analysis of the hASCT2 structure in complex with the inhibitor revealed that *Lc*-BPE is bound to the substrate binding site of ASCT2 (24). The binding site is formed by residues from HP1, TM7, and TM8 and is occluded by the extracellular gate, HP2, as depicted in Figure 1*A*. The *Lc*-BPE-bound structure additionally showed a distinctive motion of HP2 within the binding region of the outward-open conformation, relative to the glutamine-bound structure. In contrast to the closed, outward-facing conformation observed in the structure of Asp-bound EAAT1 (28), where the Na2 site is occupied (Fig. 1*B*), the Na2 site appears to be unoccupied when *Lc*-BPE binds to ASCT2. The residues that interact with *Lc*-BPE include S353 in HP1, D464, N471, and T468 in TM8, as well as other residues.

It is likely that this predicted difference in Na^+^ binding site occupancy between *Lc*-BPE-bound form of ASCT2 and the substrate bound form of EAAT1 manifests itself in the [Na^+^]-dependence of substrate/inhibitor interaction, which we tested using electrophysiological experiments. It has been previously established that competitive inhibitors, such as *Lc*-BPE, block the tonic ASCT2 leak anion conductance (9). This block results in inhibition of tonic inward current when permeable thiocyanate SCN^–^ is present inside the cell (Fig. 1*C*, black currents). Block of the leak anion current by *Lc*-BPE was Na^+^ dose dependent, with an apparent *K*_m_ for Na^+^ of 0.04 ± 0.003 mM (Fig. 1*D*), confirming previous reports of high affinity Na^+^ binding, most likely to the Na1/Na3 site(s) (29). To test the involvement of these two Na^+^ binding sites, we determined the *K*_m_ for Na^+^ of mutant transporters with a mutation to a conserved residue in the Na1 site (D473N) or Na3 site (D386N). While this *K*_m_ was virtually unchanged for the Na3 site mutant transporter, relative to ASCT2_WT_, it was significantly increased (lower apparent affinity) for ASCT2_D473N_ (Na1 site mutant, Fig. 1*E*). Moreover, the *Lc*-BPE apparent affinity was significantly decreased compared to the WT transporter in both mutants. However, this decrease was significantly more pronounced for ASCT2_D473N_ than for ASCT2_D386N_ (Fig. 1*F*). When applying serine to ASCT2_D473N,_ no significant anion current was observed, in contrast to ASCT2_D386N_ (Fig. S1*A*). Together, these results suggest that occupation of the Na1 site, but not the Na3 site is critical for *Lc*-BPE interaction with ASCT2.

To establish a comparison with the Na^+^ binding process in the presence of transported substrate, we next determined the [Na^+^] dependence of serine-induced current. Typical anion currents obtained from ASCT2 at varying concentrations of Na^+^ are shown in Figure 1*G*. At low Na^+^ concentration, serine blocked the leak conductance, resulting in reduced outward current due to the inhibition of SCN^−^ outflow (20). When Na^+^ concentration was higher than 3 mM, the current reversed to become inwardly directed, which can be attributed to the serine-induced activation of the anion conductance. Therefore, the serine-induced anion current demonstrates a biphasic dependence on the concentration of Na^+^, with a high apparent affinity observed at low [Na^+^] (*K*_m1_ = 0.04 ± 0.02 mM) and a lower apparent affinity observed at high [Na^+^] (*K*_m2_ = 49 ± 2 mM) (Fig. 1*H*). This observed biphasic Na^+^ concentration dependence also indicates that the binding of transported substrate leads to the occupation of at least two sodium-binding sites.

### The stability of bound *Lc*-BPE in MD simulations is affected by the occupation state of the Na1 and Na3 binding sites

**Figure 2:**
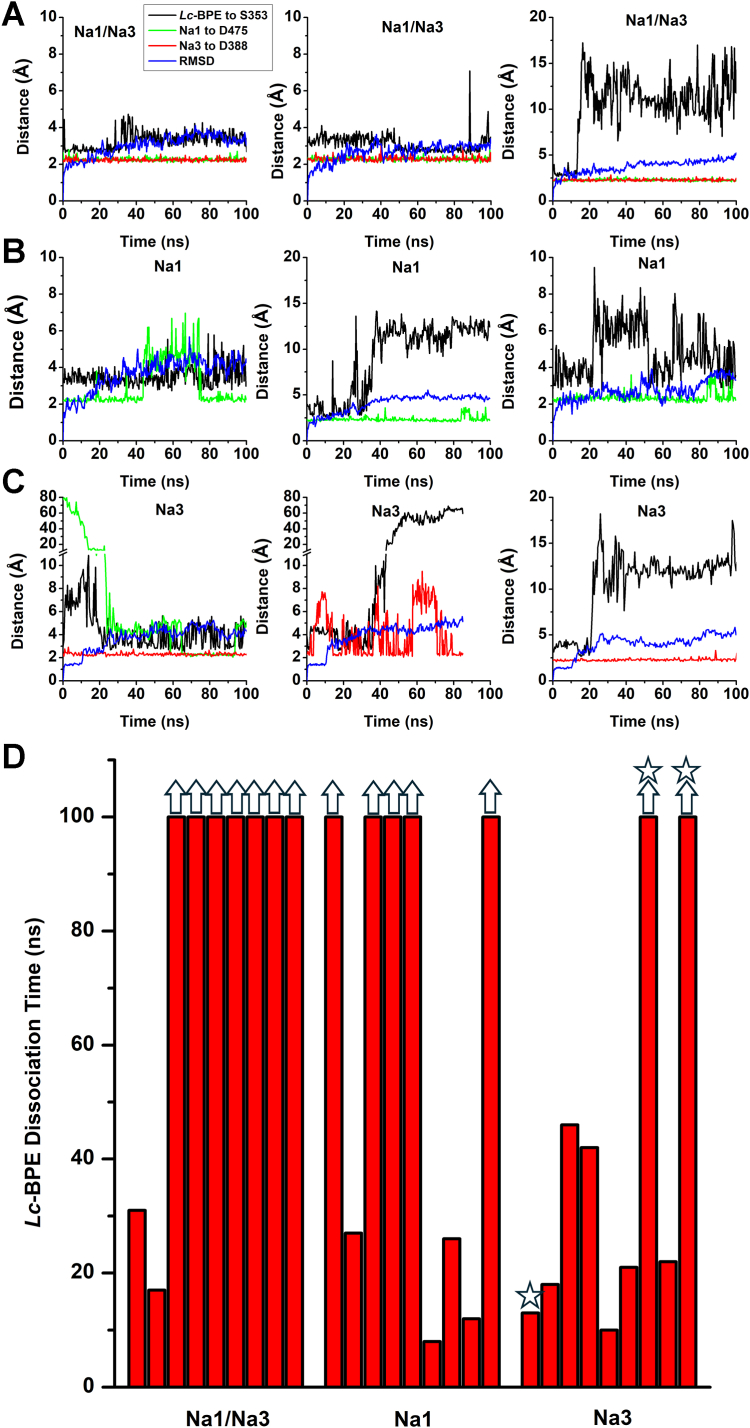
**The stability of bound *Lc*-BPE in MD simulations is affected by the occupation state of the Na1 and Na3 binding sites.***A*, representative trajectories for three independent simulations runs for *Lc*-BPE/Na1/Na3. *B*, representative trajectories for three independent simulations run for *Lc*-BPE/Na1. *C*, representative trajectories for three independent simulations run for *Lc*-BPE/Na3. Distances to selected atoms within the binding site for *Lc*-BPE (*black*), Na1 (*green*), Na3 (*red*) (RMSD is shown in *blue*). Distances were calculated based on atoms described in Experimental procedures. *D*, the residency times of *Lc*-BPE until dissociation. When distance was larger than 10 Å, the *Lc*-BPE was considered to be dissociated from the binding site. The *arrow* indicates that dissociation did not occur during the simulation of 100 ns. The *star* indicates an additional Na^+^ ion from the solution bound to the Na1 site. *Lc*-BPE, *L*-*cis* hydroxyproline biphenyl ester; MD, molecular dynamics.

To further examine the occupancy of Na^+^ binding sites in the *Lc*-BPE bound state, we performed molecular dynamics (MD) simulations with either one or two Na^+^ ions bound. We utilized the ASCT2-*Lc*-BPE complex structure (7BCS) and the EAAT1 structure with three Na ions bound (7AWM) to generate the models of ASCT2. Three models of ASCT2 were generated, each representing different binding components: *Lc*-BPE bound with Na1, Na3, or Na1/Na3 sites occupied. The Na2 site is distorted in the inhibitor-bound state, and, therefore, was not occupied in MD simulations. Subsequently, each of these models was individually inserted into a lipid-bilayer within a water box. During the 100 ns simulations of *Lc*-BPE/Na1/Na3, full dissociation of *Lc*-BPE occurred in two out of the nine simulations, but no dissociation of Na^+^ ions was observed (Figs. 2, *A* and *D* and S2). The distances between Na1, Na3, and selected residues ranged from 2 to 5 Å. During the 100 ns simulations of *Lc*-BPE/Na1, full dissociation of *Lc*-BPE occurred in four out of nine simulations, whereas one simulation showed partial dissociation of Na1 (Figs. 2, *B* and *D* and S3). During the simulations of *Lc*-BPE/Na3, full dissociation of *Lc*-BPE occurred in seven out of the nine simulations, and no dissociation of the Na^+^ ion at the Na3 site was observed (Figs. 2, *C* and *D* and S4). Remarkably, in the *Lc*-BPE/Na3 model, it has been observed that an additional Na^+^ ion from the solution spontaneously binds to the Na1 site at 48 ns, 34 ns, and 24 ns, while one of these three *Lc*-BPE remains in the binding site (Figs. 2*C* and S4). However, the *Lc*-BPE/Na1 model did not exhibit any observable binding of additional Na^+^ ions. For all simulations, there was a marginal initial rise in the RMSD, indicating a relaxation of the structure compared to its initial state. The findings collectively support the conclusion from experiment that occupancy of the Na3 site is less critical for *Lc*-BPE binding than that of the Na1 site.

### *Lc*-BPE slows recovery of inward transient current induced by substrate application

**Figure 3:**
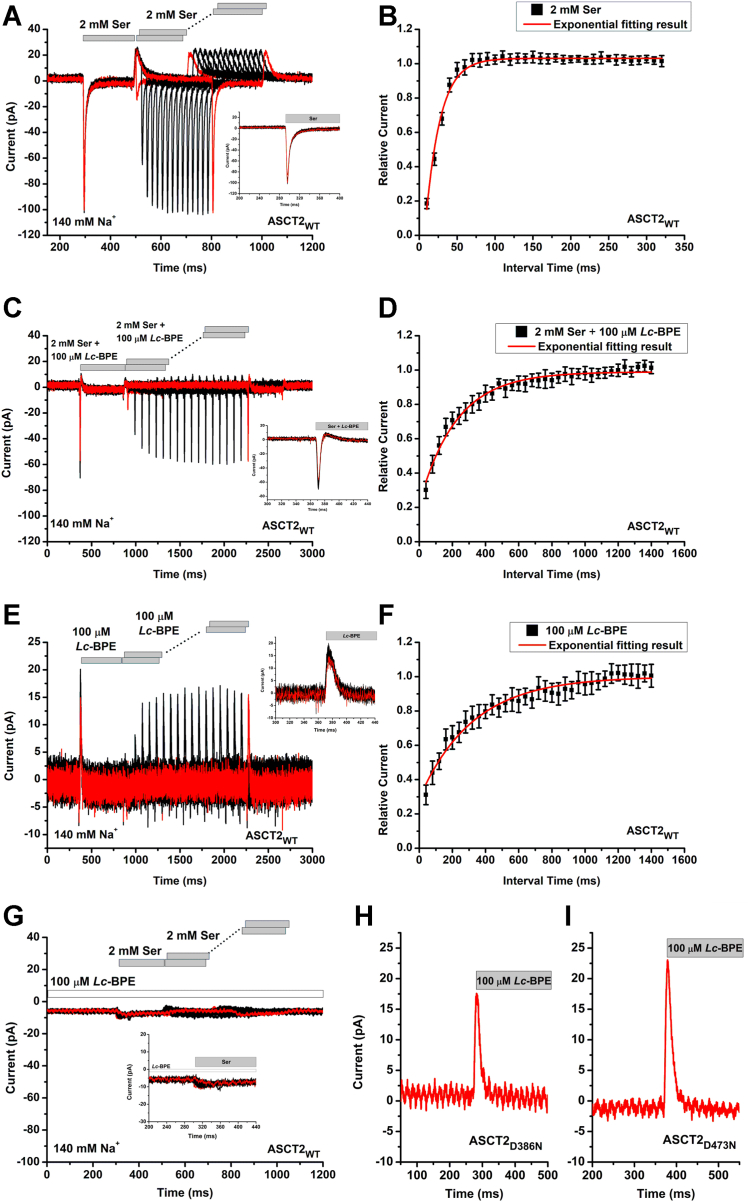
***Lc*-BPE binding slows substrate turnover and is associated with outward charge movement.***A*, transient currents recorded in ASCT2 in response to two consecutive pulses of 2 mM serine, (*C*) 2 mM serine and 100 μM *Lc*-BPE or (*E*) 100 μM *Lc*-BPE alone in the presence of 140 mM of Na^+^, with varying interpulse interval (pulse protocol shown at the *top*, *gray bars*, homoexchange conditions). The inset shows the time course of the transient current on a magnified time scale. The intracellular solution contained 130 mM NaMes/10 mM serine, the extracellular solution contained 140 mM NaMes. *B*, recovery of the transient current in the presence of 2 mM Ser, (*D*) 2 mM serine and 100 μM *Lc*-BPE or (*F*) 100 μM *Lc*-BPE alone for ASCT2_WT_. The *red solid lines* represent the best fits to an exponential equation with time constant of 19 ± 2 ms (B, serine), 230 ± 20 ms (D, serine + *Lc*-BPE) and 320 ± 60 ms (F, *Lc*-BPE alone). *G*, similar experiment as in (*C*) but in the continuous presence of 100 μM *Lc*-BPE (as shown by the *white bar*). *H*, the transient current recorded in ASCT2_D386N_ in response to 100 μM *Lc*-BPE. *I*, the transient current recorded in ASCT2_D473N_ in response to 100 μM *Lc*-BPE. The membrane potential was 0 mV in all experiments. ASCT, alanine serine cysteine transporter; *Lc*-BPE, *L*-*cis* hydroxyproline biphenyl ester.

The kinetics of the reaction steps related to the substrate translocation of ASCT2 and other transporters have been previously investigated by using rapid kinetic methods, *i.e.* laser photolysis of caged-compounds and piezo-based, rapid solution exchange (30, 31, 32, 33, 34, 35, 36). Here, to investigate the effect of *Lc*-BPE on the transport kinetics of ASCT2, we applied *Lc*-BPE rapidly using the piezo-based solution exchange system. This system allows the rapid application, as well as removal of amino acid or inhibitor, providing information on the recovery of current after amino acid and/or inhibitor removal. The kinetics of this recovery most likely reflects the turnover time of the transporter (37). The piezo-based system has a time resolution of 5 to 10 ms, which is sufficient to determine the turnover rate of ASCT2 and how it is influenced by the presence of inhibitor. As depicted in Figure 3*A*, rapid application of 2 mM serine to the extracellular side of cell membrane resulted in inward transient currents (transport component, no permeating anion present), attributed to the equilibration of the amino acid exchange equilibrium. Consistent with ASCT2’s function as an exchanger, no steady-state current was observed after the initial transient current decay (Fig. 3*A*). Upon removal of serine, an outward transient current was observed, indicative of restoration of the initial, outward facing transporter conformation. Following this first serine pulse (200 ms duration), a second pulse of serine was applied with varying delay times. In this process, the transporters have to recycle their binding sites to outward facing, completing a full transport cycle, before serine can induce another current response. The currents exhibited a relatively rapid recovery after removal of serine, as illustrated in Figure 3*B*, with a time constant of τ = 19 ± 2 ms. For ASCT2 mutants, when serine was rapidly applied to ASCT2_D386N_ and ASCT2_D473N_, the inward transient currents were still detected, but the current amplitude decreased significantly in both mutants (Fig. S1*B*).

When serine was applied simultaneously together with a saturating concentration of 100 μM *Lc*-BPE, the amplitude of the transient current was reduced to 60% of that observed in the absence of *Lc*-BPE, as depicted in Figure 3*C*. Furthermore, the rapid decay of the inward transient current was followed by a small, but significant outward overshoot, presumably due to *Lc*-BPE binding and its subsequent inhibition of the serine response (Fig. 3*C*, inset). Analysis of the recovery time of the peak current revealed a time constant of 230 ± 20 ms (Fig. 3*D*), indicating a significant increase (about 10-fold) in recovery time, and, therefore, decrease in the turnover rate compared to the absence of *Lc*-BPE. In the continuous presence of *Lc*-BPE, as depicted in Fig. 3*G*, in contrast to Figure 3*C*, the transient current was completely abolished, indicating that binding of *Lc*-BPE to the transporter is slower than that of serine. All together, these results indicate that *Lc*-BPE exhibits inhibitory effects on both presteady-state current amplitude and turnover of serine exchange. The slow turnover rate in the presence of *Lc*-BPE can be attributed to the slow dissociation of the inhibitor (see below).

### *Lc*-BPE binding to ASCT2 is associated with outward charge movement

Next, we applied *Lc*-BPE in the absence of substrate; upon the application of *Lc*-BPE, outward transient currents were observed (Fig. 3*E*). This result was unexpected, due to *Lc*-BPE being a neutral amino acid derivative, which should not carry a net charge. The time constant for transient current recovery was found to be 320 ± 60 ms (Fig. 3*F*), which is consistent with the time scale of recovery observed for the simultaneous application of serine and *Lc*-BPE (Fig. 3*D*). These results indicate that *Lc*-BPE demonstrates a slow rate of dissociation from the transporter binding site. To initiate the serine-induced inward transient current after *Lc*-BPE removal, the substrate needs to displace *Lc*-BPE, which has to dissociate first. Therefore, the simultaneous application of serine and *Lc*-BPE results in the rate of recovery being determined mostly by inhibitor dissociation rather than substrate-induced turnover. For the Na1 and Na3 site mutant transporters, the outward transient currents were still observed when *Lc*-BPE was rapidly applied to ASCT2_D386N_ and ASCT2_D473N_ and the current amplitude remained relatively unchanged (Figs. 3, *H* and *I* and S1*B*).

### Na^+^ dependency of *Lc*-BPE-induced transient current is consistent with the occupation of Na1/Na3 sites, but not the Na2 site

**Figure 4:**
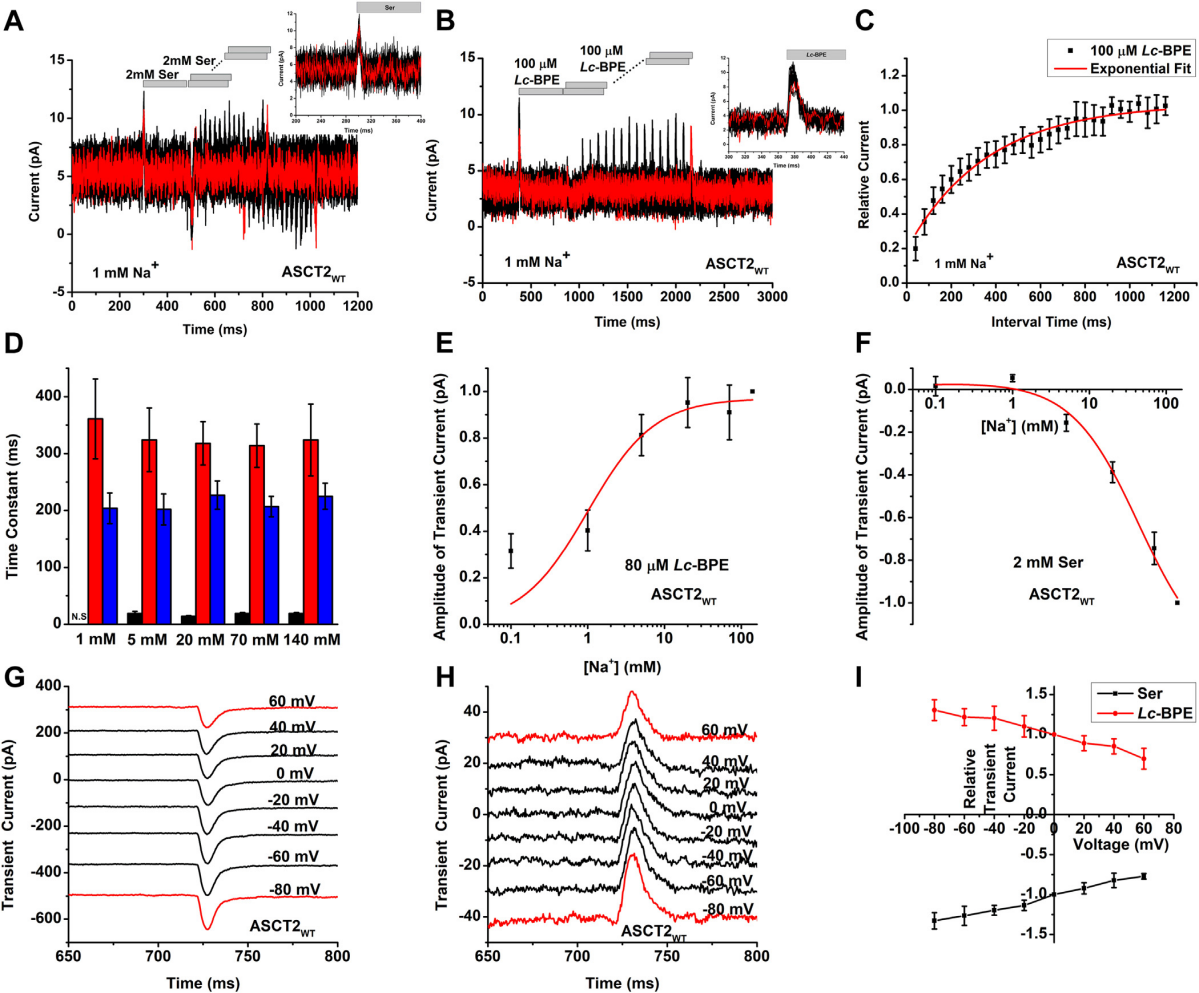
**Outward transient currents in response to *Lc*-BPE are consistent with occupation of high affinity Na**^**+**^**binding site(s) and show weak voltage dependence.***A*, transient currents of ASCT2 in response to two pulses of rapid 2 mM serine, or (*B*) 100 μM *Lc*-BPE application in the presence of 1 mM Na^+^, with varying interpulse interval (pulse protocol shown at the top) under homoexchange conditions. The intracellular solution contained 130 mM NaMes/10 mM serine, the extracellular solution contained 140 mM NaMes. The inset shows the time course of the transient current on a magnified time scale. *C*, recovery of the transient current in the presence of 100 μM *Lc*-BPE alone at 1 mM Na^+^. The *red solid lines* represent the best fits to an exponential equation with time constant of 360 ± 70 ms. The membrane potential was 0 mV in all experiments. *D*, time constant of transient current recovery of 2 mM serine (*black*) or 100 μM *Lc*-BPE (*red*) application in the presence of 1/5/20/70/140 mM Na^+^ or 2 mM serine + 100 μM *Lc*-BPE (*blue*) in the presence of 140 mM Na^+^. *E*, amplitude of transient current induced by *Lc*-BPE application as a function of [Na^+^]. *F*, amplitude of transient current induced by serine application as a function of [Na^+^]. The *red solid lines* represent the best nonlinear curve fit to a Michaelis–Menten-like equation. The apparent affinities for Na^+^ were calculated as *K*_m_ = 1.0 ± 0.5 mM for (E, *Lc*-BPE), *K*_m1_ = 0.04 ± 0.4 mM, *K*_m2_ = 44 ± 20 mM for (F, serine). *G*, typical serine-induced transient current recordings at varying membrane potentials (from −80 to + 60 mV, [serine] was 2 mM). *H*, similar experiment as in (*G*) but for *Lc*-BPE. *I*, the amplitude of transient current as function of the membrane potential for application of serine and *Lc*-BPE. ASCT, alanine serine cysteine transporter; *Lc*-BPE, *L*-*cis* hydroxyproline biphenyl ester.

To test if the outward transient current observed upon the application of *Lc*-BPE is coupled to the binding of Na^+^ ions, the rapid kinetic experiments were performed using varying concentrations of Na^+^. Typical currents recorded in the presence of 70 mM, 20 mM, 5 mM, and 1 mM Na^+^ are depicted in Figs. S5–S7, and Figure 4*B*, respectively. In the presence of serine, the transient currents exhibited an inward direction at Na^+^ concentrations of 70 mM, 20 mM, and 5 mM, as depicted in Figs. S5*A*, S6*A* and S7*A*. As expected, the amplitude of serine-induced current was significantly decreased when the concentration of Na^+^ was reduced from 140 mM to 5 mM (Fig. 4*F*). However, the recovery time of transient current was unaffected by the Na^+^ concentration (Fig. 4*D*). In contrast, at Na^+^ concentrations of 1 mM and below, the currents reversed sign toward the outward direction, as depicted in Figure 4, *A* and *F*, reminiscent of that observed after application of *Lc*-BPE.

For the transient current induced by *Lc*-BPE, in contrast to the results with serine, currents exhibited an outward direction at all Na^+^ concentrations (Figs. S5*C*, S6*C*, S7*C* and 4*B*). The amplitude of the currents was virtually independent of [Na^+^] at concentrations >5 mM (Fig. 4*E*). However, a notable decrease in amplitude was seen when the concentration was reduced to 1 mM, as depicted in Figure 4, *B* and *E*. The recovery time of the transient current was not affected across all Na^+^ concentrations (Fig. 4*D*). The apparent *K*_m_ value for Na^+^ was determined to be 1.0 ± 0.5 mM for *Lc*-BPE, as calculated from the transient current (Fig. 4*E*), consistent with the high affinity observed for the Na^+^ binding to the *apo*-form of the transporter (most likely Na1 and Na3 sites), suggesting that the binding of *Lc*-BPE is solely associated with occupation of the *apo* transporter binding site(s) with Na^+^, but does not require Na^+^ binding to the *Lc*-BPE-bound form (presumably Na2 site).

Next, we investigated the voltage dependence of the transient currents in the presence of serine or *Lc*-BPE; the experiments were conducted at varying voltages from −80 to +60 mV (Fig. 4, *G* and *H*). Transient currents were then plotted as a function of the membrane potential, as shown in the I-V curves in Figure 4*I*. Both the serine-induced inward transient current and the *Lc*-BPE-induced outward transient current were weakly voltage dependent, exhibiting a small but significant increase as the membrane potential became more negative. This observation is in consistent with the hypothesis that binding of Na^+^ prior to serine or *Lc*-BPE is voltage dependent, as proposed previously (29), leading to an increase in affinity of serine or *Lc*-BPE.

### *Lc*-BPE slows the onset of anion currents

**Figure 5:**
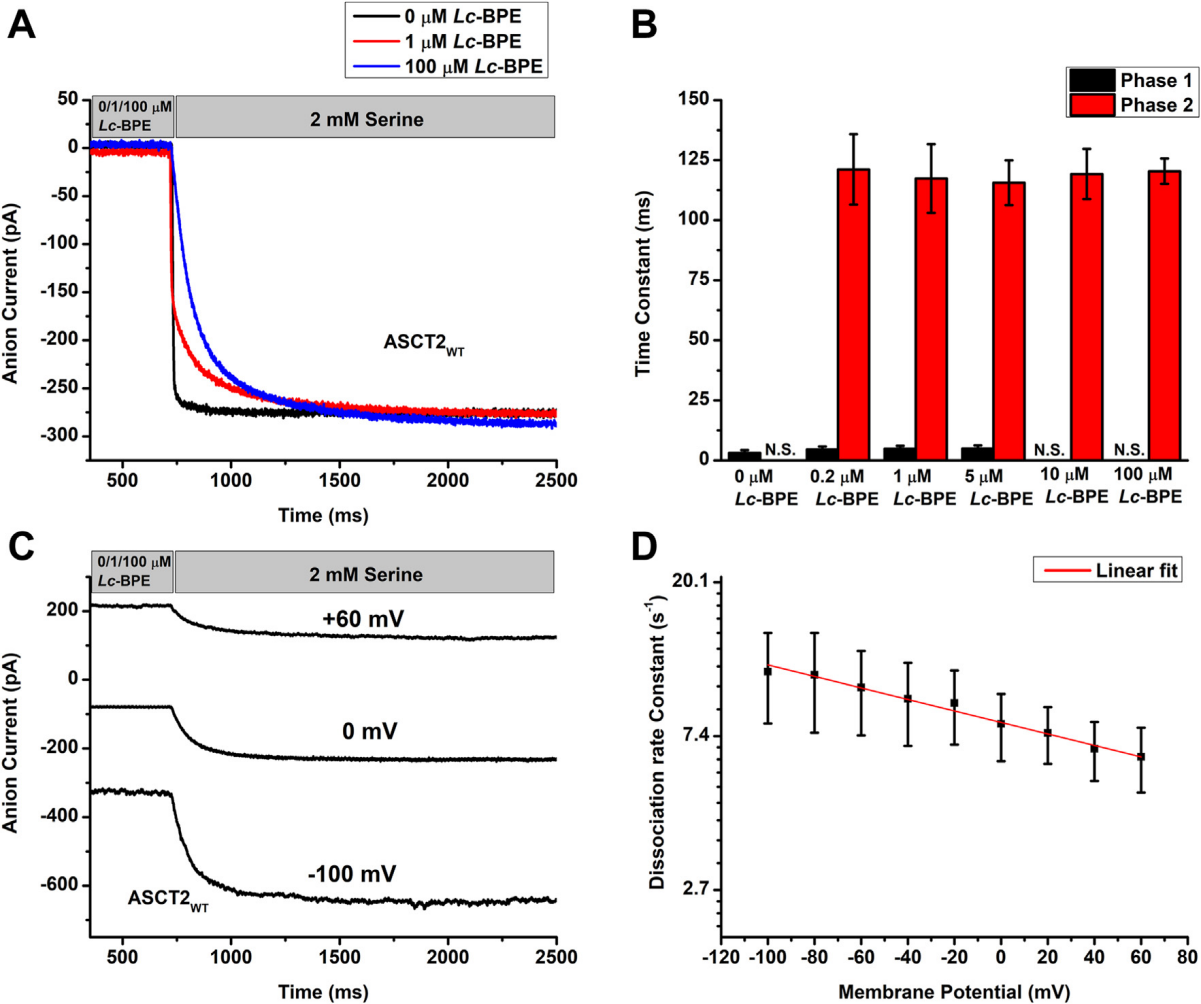
***Lc*-BPE dissociation rate constant is independent of *Lc*-BPE concentration but dependent on the membrane potential.***A*, typical traces of whole-cell current recordings of ligand displacement experiments. Cells were preincubated with buffer only (*black trace*), 1 μM *Lc*-BPE (*red trace*) or 100 μM *Lc*-BPE (*blue trace*). Serine (2 mM) was rapidly applied through solution exchange to ASCT2. (The application time for *Lc*-BPE and serine is indicated by the *gray bar*). The intracellular solution contained 130 mM NaSCN/10 mM serine, the extracellular solution contained 140 mM NaMes. *B*, *Lc*-BPE dissociation time constants in the presence of increasing concentrations of *Lc*-BPE. A two-exponential equation was used to fit the presteady state for 0.2 μM, 1 μM, and 5 μM *Lc*-BPE and a single-exponential equation for 0 μM, 10 μM, and 100 μM *Lc*-BPE upon substrate application (see Experimental procedures). *C*, typical currents at 0 mV, −100 mV or +60 mV membrane potential. *D*, *Lc*-BPE (100 μM) ln of the dissociation rate constant as a function of the cell membrane potential (linear fit, slope was −0.0037 ± 0.0002/mV). ASCT, alanine serine cysteine transporter; *Lc*-BPE, *L*-*cis* hydroxyproline biphenyl ester.

To evaluate the inhibition kinetics, we determined the dissociation rate of *Lc*-BPE using a ligand displacement assay. Here, the ASCT2-expressing cells were preincubated with a certain concentration of *Lc*-BPE in the presence of intracellular SCN^−^ (recording the anion current component). Following preincubation, *Lc*-BPE was rapidly replaced with 2 mM serine, which saturated the substrate binding site. After preincubation with *Lc*-BPE, a significant slowing of serine-induced anion current rise was observed (Fig. 5*A*). At 0.2 μM, 1 μM, and 5 μM preincubation, the substrate-induced anion current rise was biphasic, with a rapidly rising phase (phase 1) and a slow-rising phase (phase 2), presumably corresponding to the fraction of the transporter without or with *Lc*-BPE bound before serine application. The biphasic current was then fitted with two exponential components, the apparent time constants in 1 μM *Lc*-BPE preincubation were 4.9 ± 1.2 ms for phase 1 and 120 ± 14 ms for phase 2 (Fig. 5, *A* and *B*). In the absence of *Lc*-BPE, only the rapidly rising phase was observed. This phase had a time constant of 3.1 ± 1.2 ms, which is in line with phase 1 time constant observed in the presence of *Lc*-BPE. Subsequently, we further investigated *Lc*-BPE dissociation at even higher concentrations. At a concentration of 10, 100 μM, only the slow-rising phase was seen, indicating that the binding site for *Lc*-BPE was fully occupied before serine displacement (Figs. 5, *A* and *B*). As expected for inhibitor displacement, which is rate-limited by *Lc*-BPE dissociation, the time constants for both phases were independent of the *Lc*-BPE concentration (Fig. 5*B*).

### The time constant for *Lc*-BPE dissociation is sensitive to the membrane potential

Next, the time constant for *Lc*-BPE dissociation was quantified as a function of the transmembrane potential, using the same ligand displacement assay. This time constant decreased weakly with more negative membrane potential, indicating faster dissociation (Fig. 5, *C* and *D*). Based on the observed slope of the ln(*k*) *versus* voltage relationship in Fig. 5*D*, the valence of the *Lc*-BPE binding, *z*_Q_, was determined to be −0.19 ± 0.01 based on equation (1):

(1) $$k\;\left(V\right)=k\left(0\;mV\right)\exp\left(\frac{z_{Q}FV}{2RT}\right)$$

Here, V is the membrane potential, R is the gas constant, T the temperature, and F the Faraday constant. This result is consistent with an outward movement of negative charge or an inward movement of positive charge upon *Lc*-BPE dissociation (opposite of the charge movement from binding determined above), which are expected to accelerate at negative membrane potential. This observation suggests that the binding/dissociation of *Lc*-BPE is influenced by the electrical potential across the cell membrane, thereby further characterizing them as weakly electrogenic processes.

### Prediction of electrostatic contributions to substrate/inhibitor binding mechanism

**Figure 6:**
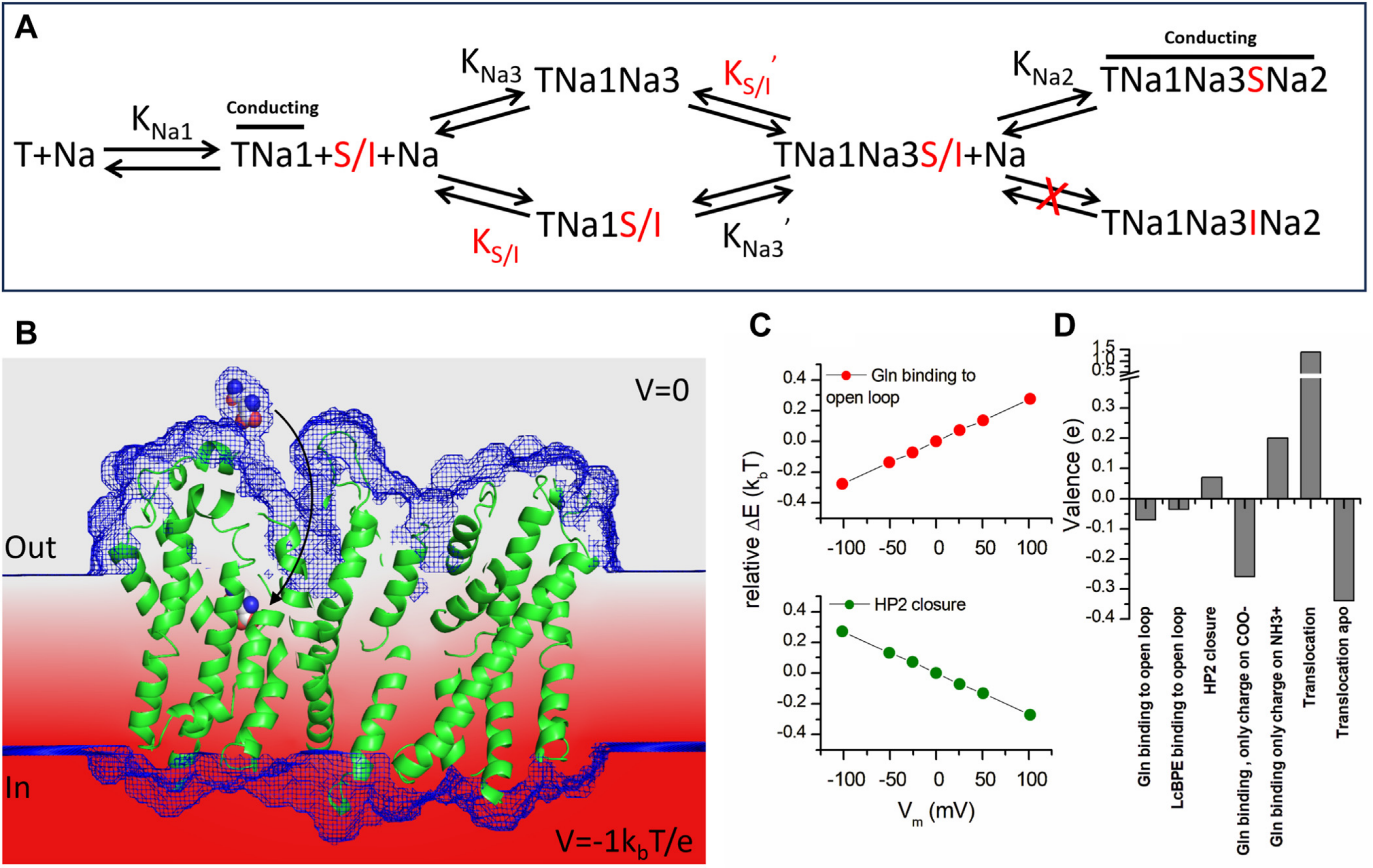
**Proposed simplified mechanism of competitive inhibitor (In) binding to transporter (T).***A*, the amino acid substrate is abbreviated as S, the inhibitor as I. The bars illustrate the anion-conducting states. *B*, illustration of the setup of the electrostatic calculations of the valence of the charge movement caused by glutamine (atoms represented as *spheres*) binding (*arrow*). The protein backbone is shown in *green* (PDB code: 6mpb), the accessibility map calculated using APBSMem is shown as the *blue mesh*, the membrane potential gradient calculated using a noncharged transporter is shown in *red*. *C*, relative energy difference between two states of the system for gln binding (unbound and bound state, *top*) and HP2 closure (in absence of gln, *bottom*) as a function of the membrane potential. The valence for the transition, (*D*) is obtained from the slope of the relationships shown in (*C*). HP2, hairpin loop 2; PDB, Protein Data Bank.

To further determine the physical basis behind the outward charge movement caused by application of neutral, but zwitterionic amino acid to ASCT2, we performed calculations of the electrostatics of the binding reaction. This approach is based on the solving of a linearized Poisson-Boltzmann equation using the Adaptive Poisson-Boltzmann Solver, APBS (https://apbs.readthedocs.io/en/latest/index.html), with the APBSMem protocol that is based on the placement of the protein in an implicit membrane and application of a membrane potential (38, 39). Calculation of the electrostatic energy change when switching between two states of the system (for example *apo* to amino acid bound) at varying membrane potentials allows the calculation of the valence associated with a potential charge movement. The setup of the system and calculation of the valence are illustrated in Figure 6, *B* and *C*. As expected, translocation of the fully loaded transporter is associated with a positive valence (Fig. 6*D*), due to the inward movement of net positive charge associate with the bound Na^+^ ions. In contrast, the cation binding sites of the empty transporter are predicted to carry negative charge (Fig. 6*D*). Interestingly, glutamine binding to the outward open-loop configuration of ASCT2 is predicted to have a valence of −0.08, consistent with transient outward current induced by substrate binding. A similar, negative valence was calculated for *Lc*-BPE binding. In contrast, HP2 closing is associated with a small positive valence (Figs. 6, *C* and *D*). Where could this negative charge movement upon binding originate from? A possible explanation would be that the negative charge of the α-carboxy group of the amino acid is more deeply buried in the membrane compared to the positive charge of the α-amino function. Consistent with this hypothesis, the valence of binding of a glutamine molecule, in which all charges but one negative charge on the carboxy group were neutralized, was −0.26. In contrast, the absolute valence of binding with only one positive charge on the amino group was smaller, *z* = +0.20 (Fig. 6*D*). Together, these results suggest that the negative carboxy group moves deeper into the membrane electric field than the amino group, thus generating an overall negative valence for substrate/inhibitor binding. On the other hand, occlusion of the negative charge of the binding site is predicted to be associated with a very small valence (about −0.01). Overall, the results of the electrostatic calculations are consistent with the experimental results, showing outward charge movement induced by both amino acid and *Lc*-BPE application.

### *Lc*-BPE application has only minor effect on the glutamate transporter

**Figure 7:**
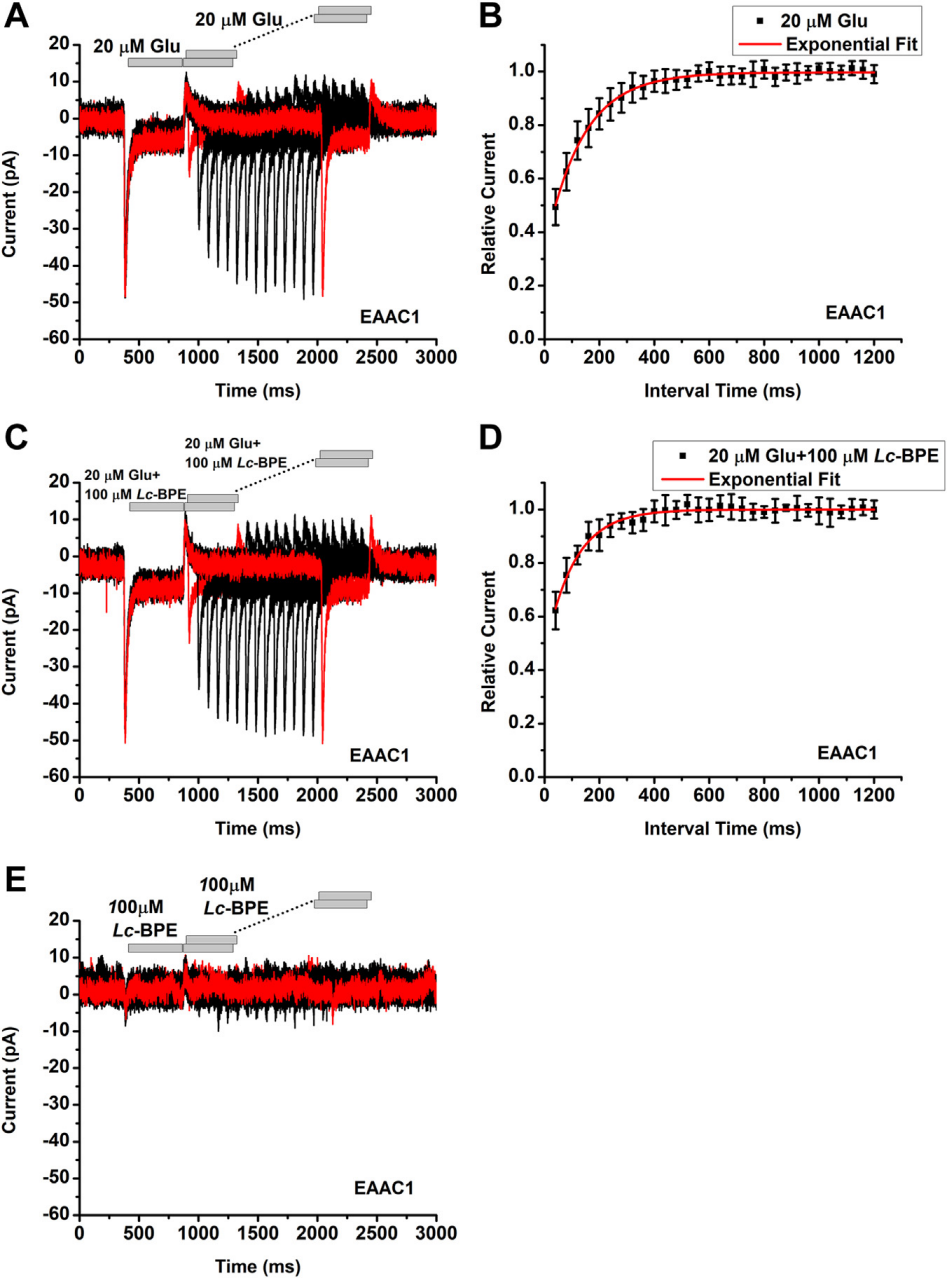
***Lc*-BPE has minor effect on substrate translocation in EAAC1.***A*, transient currents recorded in EAAC1 in response to two consecutive pulses of 20 μM glutamate, (*C*) 20 μM glutamate and 100 μM *Lc*-BPE or (*E*) 100 μM *Lc*-BPE alone application in the presence of 140 mM of Na^+^, with varying interpulse interval (pulse protocol shown at the *top*, *gray bars*). The intracellular solution contained 130 mM KMes, the extracellular solution contained 140 mM NaMes. *B*, recovery of the transient current in the presence of 20 μM glutamate or *D*, 20 μM glutamate and 100 μM *Lc*-BPE. The *red solid lines* represent the best fits to an exponential equation with time constant of 140 ± 8 ms (glutamate) and 110 ± 10 ms (glutamate + *Lc*-BPE). *E*, application of 100 μM *Lc*-BPE alone in the presence of 140 mM of Na^+^. The membrane potential was 0 mV in all experiments. EAAC1, excitatory amino acid carrier 1; *Lc*-BPE, *L*-*cis* hydroxyproline biphenyl ester.

It was reported that *Lc*-BPE also inhibits excitatory amino acid carrier 1 (EAAC1), the rat analog of human EAAT3, although with lower apparent affinity (24). Glutamate transport by EAAC1 is electrogenic, involving the cotransport of three Na^+^ ions and one proton, as well as the counter transport of one K^+^ ion, for each transported glutamate molecule. Consistent with this stoichiometry, large inward transient currents were observed upon rapid application of glutamate, followed by a steady-state current component caused by electrogenic transport (Figs. 7*A* and S8, *A* and *C*). The time constant for transient current recovery was calculated to be 140 ± 8 ms (Fig. 7*B*). Recovery of the transient current was slowed and peak amplitudes were reduced in the presence of 100 μM DL-TBOA (40), a competitive inhibitor specifically targeting glutamate transporters (Fig. S8, *A* and *C*). TBOA application alone (without glutamate) resulted in a small, transient outward current, followed by an inwardly directed overshoot current (Fig. S8*E*), consistent with previous results with kainate, another glutamate transporter inhibitor (41). These results were as expected for competitive inhibitor behavior with EAAC1. In contrast to TBOA, *Lc*-BPE had virtually no effect on the recovery time constant of the transient current (110 ± 10 ms, Fig. 7, *C* and *D*). Furthermore, in contrast to ASCT2, the application of *Lc*-BPE alone did not induce significant transient current in EAAC1 (Fig. 7*E*). Together, these results indicate that while *Lc*-BPE may be able to inhibit EAAC1 anion current, it has much less effect on stoichiometric charge movement by the transporter.

## Discussion

In this report, we detail the involvement of electrostatics in the binding mechanism of competitive inhibitors, substrates as well as Na^+^ ions in the glutamine transporter ASCT2, including a comprehensive analysis of results from electrophysiological and rapid kinetic experiments. First, our results indicate that *Lc*-BPE binding is consistent with a model that only requires Na^+^ binding to the *apo*-form of the transporter. In this model, at least one or two Na^+^ ion(s) initially bind(s) to the transporter when it is unoccupied (presumably to the Na1/Na3 sites), resulting in the formation of a high-affinity inhibitor/substrate binding site (Fig. 6*A*). Results from mutagenesis and MD simulations indicate that the Na1 site, rather than the Na3 site, plays a more critical role in *Lc-*BPE binding, although occupancy of both Na1 and Na3 sites resulted in the best ligand stability in MD simulations. Second, in contrast to transported substrate, HP2 cannot fully close in the *Lc*-BPE-bound state; thus, the Na2 site is not formed and cannot be occupied. Third, initial binding of *Lc*-BPE and substrate are associated with outward charge movement, despite these compounds having a net neutral charge. Fourth, *Lc*-BPE not only suppresses the substrate-induced anion current, but also slows the kinetics of the serine-induced current and the turnover of serine exchange. Finally, displacement experiment suggests that the rate of activation of anion current induced by substrate is slowed when the cell was preincubated with *Lc*-BPE, pointing to relatively slow *Lc*-BPE dissociation kinetics, *i.e.* long residency time of inhibitor in the binding site.

The sequence of sodium and amino acid binding to the transporters is conserved in the SLC1 family (29, 33, 42, 43). Amino acid transport requires the cotransport of two or three Na^+^ ions. It was proposed that in the *apo*-state of the transporters, HP2 is closed, preventing access to the substrate binding site (19). Subsequently, in ASCTs, Na^+^ binds to Na1/Na3 sites with very high apparent affinity in the submillimolar range, which is consistent with our result in Figure 1. This is one of the major functional differences between the ASCT and EAAT members of the family, where in the EAATs initial Na^+^ binding is of much lower apparent affinity, in the 100 mM range. The binding to and occupation of the Na1/Na3 sites induces remodeling of the substrate binding site, leading to the opening of HP2 and facilitating substrate binding. This process is accompanied by the closure of HP2, which effectively secures the substrate in the outward-occluded state (16, 17, 18). On the other hand, binding of the competitive inhibitor *Lc*-BPE prevents loop closure, with the consequence that the third Na^+^ ion is unable to bind to the Na2 site. The cryo-EM structure of *Lc*-BPE-hASCT2 showed that HP2 is less resolved, suggesting the HP2 gate exhibits flexibility and has the capacity to move further to accommodate bulkier inhibitors (24), thus disrupting the structure of the Na2 site. The result is that *Lc*-BPE binding cannot induce the inward charge movement seen upon application of transportable substrates, which is indicative of Na^+^ binding to the Na2 site and structural changes following this binding. The likely sequence of binding of Na^+^ and substrate/inhibitor, accounting for the experimental data for WT and Na-site mutant transporters is illustrated in the mechanism shown in Figure 6*A*.

Results from the rapid kinetic experiments also indicate that the presence of *Lc*-BPE leads to a complete inhibition of serine-induced inward transient charge movement, as well as a substantial decrease in the turnover rate of the transporter, as expected for a competitive inhibitor. This is evident from the observed >10-fold reduction in the recovery rate of transient current after substrate removal (Fig. 3). Due to the long residency time of *Lc*-BPE in the binding site (see below) the recovery kinetics should depend primarily on the inhibitor dissociation rate rather than the substrate turnover rate. On the other hand, rapid application of *Lc*-BPE alone induced outward transient currents. In some experiments, as depicted in Figure 3*E*, discernible inward overshoots were detected, possibly resulting from the binding of Na^+^ ions to the unoccupied transporters (44). A transient outward current can generally be induced by the outward movement of positively charged ions or the inward movement of negatively charged ions, until a new state or equilibrium is reached. Due to its largely net neutral nature at physiological pH (the isoelectric point of proline is 6.3), it is, therefore, not immediately evident why *Lc*-BPE binding would result in charge movement. However, the application of serine at low Na^+^ concentrations, when the Na2 site cannot be occupied, also induced outward transient currents, hence exhibiting a similar functional effect as a competitive inhibitor. Therefore, it is unlikely that this outward current is an artifact.

What could be the molecular basis of the outward charge movement upon substrate/inhibitor binding, despite the net neutral charge of the zwitterionic species? Our results from calculations of the electrostatic interaction of the amino acid with its binding site point to the following hypothesis: The negatively charged α-carboxy group of the amino acid penetrates the membrane electric field more deeply than the positively charged α-amino group. The predicted overall valence of the charge movement is small (less than 10% of one charge), consistent with the weak voltage dependence of the dissociation time constant. This voltage dependence demonstrates that a more positive membrane potential results in a longer residency time of the ligand in the binding site, as expected, if the residency time is dominated by the membrane potential’s effect on the bound negative charge of the α-carboxy group of the ligand. In contrast, the amplitude of the substrate/inhibitor induced current shows the opposite voltage dependence, increasing with a more negative potential, indicating that this amplitude is not controlled by ligand binding, but rather by some other, voltage dependent step, possibly conformational changes of HP2.

Overall, these results open intriguing possibilities for the electrophysiological detection of ligand binding to transmembrane proteins, and/or conformational changes associated with those binding events. Even for net neutral ligands, charge movement may be detected electrophysiologically, if there is an asymmetrical charge distribution when the molecule is bound in the transmembrane electric field. While electrogenic partial reactions have been observed in the electroneutral Na/H exchanger (45) and serotonin transporter (46), both involving charged ions/substrates, to our knowledge, electrophysiological analysis of charge movements induced by neutral molecules has not been reported in the literature. Ultimately, this may be the main significance of this work, extending electrophysiological methods to membrane proteins that have not been thought to be amenable to a current recording approach. Therefore, this may have implications for the study of other important electroneutral transporters. For example, Na^+^-independent amino acid transporters, such as the large amino acid transporter, LAT1, could be reconsidered to test with an electrophysiological approach.

Are there alternative explanations for the outwardly directed charge movement upon inhibitor/substrate application? One possibility could be that less than 100% of the substrate binding sites are outward facing before *Lc*-BPE application. In other words, the translocation equilibrium is not fully pushed to the outward-facing state. Due to the zero-*trans* conditions for amino acid substrate used in these experiments (saturating [serine] inside the cell, zero [serine] on the outside), this is not likely. In addition, lowering external [Na^+^] would be expected to further push the equilibrium to outward facing, but the outward current is maintained to very low external [Na^+^]. However, if this was the case, *Lc*-BPE binding would be expected to increase the population of the outward facing state. Thus, the outward current would be caused by outward movement of the positively charged transport domain, which is loaded with amino acid and Na^+^. In a recent publication, Burtscher et al. report similar rapid transient currents induced by binding of the inhibitor cocaine to the serotonin transporter, serotonin transporter (46). Cocaine is positively charged. The binding current was attributed to the displacement of Gouy-Chapman surface charge. In contrast, the amino acid derivatives used in our report are net neutral. Therefore, the Gouy-Chapman hypothesis is less likely. Other limitations of our study are related to the reliance on functional measurements, albeit with high time resolution and sensitivity. Ultimately, the determination of subtle conformational changes that determine inward or outward charge movement will have to come from structural studies.

Our results also provide further insight into the residency time of the competitive inhibitor, *Lc*-BPE, in the binding site. The dissociation kinetics of *Lc*-BPE in displacement experiments are characterized by a slow dissociation rate. The activation rate by serine of the anion current is slowed when the cell is preincubated with the *Lc*-BPE inhibitor, reflecting the dissociation of the inhibitor. The displacement of the bound inhibitor by the substrate takes place within a time scale of 120 ms. Such a slow apparent dissociation time constant agrees with the high binding affinity exhibited by *Lc*-BPE, in the sub-μM range. In contrast, residency time for serine is much shorter, with the higher dissociation rate being a necessity for rapid transporter turnover during amino acid exchange.

To summarize, in the present study, the binding mechanism of a competitive inhibitor in ASCT2 was elucidated through the analysis of presteady-state kinetic data. Our results suggest that the binding of a competitive inhibitor is coupled to the binding of Na^+^ to the Na1/Na3 site, while the Na2 site remains unoccupied. In contrast, the binding of substrate, subsequently leading to transport, necessitates the occupancy of three Na sites, including the Na2 site. At low concentrations of sodium, the Na2 site is not occupied, even in the substrate bound form, resulting in electrophysiological behavior that is indistinguishable from that of the inhibitor, *Lc*-BPE. With respect to the actual electrostatic contribution to the substrate/inhibitor binding process, it was determined that movement of the negatively charged α-carboxy group into the transmembrane electric field dominates the voltage dependence of the process, even though the amino acid has a net neutral charge. The presence of *Lc*-BPE also resulted in a slowing of substrate exchange turnover and a decrease in the rate of activation of substrate-induced anion current, due to its long residency time in the ASCT2 binding site. Given the increasing significance of ASCT2 as a potential target for anticancer drugs, our findings have the potential to contribute to the mechanism-based development and optimization of ASCT2 inhibitors.

## Experimental procedures

### Cell culture and transfection

Human embryonic kidney 293T cells (HEK293T, American Type Culture Collection No. CRL 1573) were cultured as described previously (47, 48). rASCT2 or EAAC1 complementary DNAs constructs inserted in the pBK-CMV vector were transiently transfected into cells using jet-PRIME transfection reagent. Cells were tested for *mycoplasma* and authenticated by short tandem repeat profiling (ATCC). Transfections were performed according to the protocol supplied by Polyplus-Transfection. The cells used for electrophysiological analysis were cultured for 20 to 30 h after transfection. Mutagenesis was performed using the QuikChange protocol according to the directions of the supplier (Agilent). Mutations were confirmed using sequencing.

### Electrophysiology

Currents associated with rASCT2 and EAAC1 were measured in the whole-cell current recording configuration with an EPC7 patch-clamp Amplifier (ALA Scientific). Whole-cell currents were recorded under voltage-clamp conditions. The resistance of the recording electrode was 3 to 6 MΩ. Series resistance was not compensated because of the small whole-cell currents carried by EAAC1 and ASCT2. For ASCT2, the composition of the solutions for measuring amino acid exchange currents in the transport mode was as follows: 140 mM sodium methanesulfonate (NaMes), 2 mM Mg gluconate 2, 2 mM CaMes_2_, 10 mM Hepes, with additional amino acid substrates or/and inhibitor, pH 7.3 (extracellular), and 130 mM NaMes (for the measurement of anion current, intracellular NaMes was replaced with sodium thiocyanate (NaSCN)), 2 mM Mg gluconate 2, 5 mM EGTA, 10 mM Hepes, pH 7.3 (intracellular), as published previously (47, 49, 50). For EAAC1, the composition of the solutions for measuring amino acid exchange currents in the transport mode, the intracellular NaMes was replaced by KMes. For experiments on Na^+^ dependence, the concentration of extracellular NaMes was adjusted and compensated for by NMGMes.

### Rapid solution exchange

Fast solution exchanges were performed using the SF-77B (Warner Instruments LLC) piezo-based solution exchange instrument, allowing a time resolution in the 5 to 10 ms range. Amino acid substrate was applied through a theta capillary glass tubing (TG200-4, outer diameter = 2.00 mm, inner diameter = 1.40 mm; Warner Instruments), with the tip of the theta tubing pulled to a diameter of 350 μm and positioned at 0.5 mm to the cell (37). For paired-pulse experiments, currents were recorded with 10/20/40 ms interval time after removal of amino acid.

### Voltage-jump experiments

Voltage jumps (−100 to +60 mV) were applied to perturb the translocation equilibrium and to determine the voltage dependence of the transient current and anion conductance. To determine ASCT2-specific currents, external solution contained 140 mM NaMes in the presence of amino acid substrate and/or competitive inhibitor (as control). The internal solution contained 130 mM NaMes in the presence of 10 mM amino acid substrate (for the displacement experiment, intracellular NaMes was replaced with NaSCN). Capacitive transient compensation and series resistance compensation of up to 80% was used using the EPC-7 amplifier.

### Electrostatic calculations of Gln and Lc-BPE binding valence

We utilized the APBSmem 2.1.0 (38) (https://apbsmem.sourceforge.io/), to compute electrostatic energies based on the adaptive Poisson-Boltzmann solver, APBS (39). These calculations involved examining the binding of the inhibitor *Lc*-BPE to ASCT2 within an implicit membrane environment. Our analysis considered substrate Gln binding, along with the HP2 open and closure states. In the presence of an internal membrane potential, *V*, the following modified version of the linearized Poisson-Boltzmann equation is used, according to the procedure first introduced by Roux (51):

(2) $$\begin{array}{l} -\nabla \left[\varepsilon \left(\vec{r}\right)\nabla \varphi \left(\vec{r}\right)\right]+{\bar{\kappa }}^{2}\left(\vec{r}\right)\varphi \left(\vec{r}\right)\\ =\frac{e4\pi }{k_{b}T}\left(\rho \left(\vec{r}\right)+\frac{{\bar{\kappa }}^{2}V}{4\pi }f\left(\vec{r}\right)\right) \end{array}$$

Here, ε represents the spatially dependent dielectric constant, *Φ* is the electrostatic potential, and *κ* stands for the Debye–Hückel screening constant and *k*_b_ the Boltzmann constant. The function *f*I as a Heaviside step function, specifically set at 1 in the intracellular solution and 0 in the membrane, protein, and extracellular solution. Details can be found in reference (38).

The total electrostatic energy, *E*, is then computed by summing up over the product of the local charge and the potential (52):

(3) $$E=\int \varphi (\vec{r)}\rho (\vec{r)}dV$$

The valence is computed by applying various internal membrane potentials (*V*) and calculating the difference in total electrostatic energy Δ*E* between protein configurations with bound and free substrate. The valence is determined from the slope of the ΔE *versus* membrane potential plot (53). Further details are available in previous publications (41, 54).

### Data analysis

The data analysis was performed in Microsoft Excel (https://www.office.com/) and Microcal Origin (https://www.originlab.com/) software. Error bars are shown as mean ± SD, collected from recordings of 6 to 10 cells, for statistical analysis.

To determine the recovery rate of transient currents, nonlinear curve fitting was used with the following exponential function:

(4) I=Imax∗(1−exp(−tτ))

Here, *I* is the current amplitude, *I*_max_ is the current for the first pulse, τ the time constant, and t the time.

Transient signals of piezo-based solution-exchange results were analyzed in Clampfit software (Axon Instruments, https://www.moleculardevices.com/products/axon-patch-clamp-system/acquisition-and-analysis-software/pclamp-software-suite) by fitting with a sum of two exponential components:

(5) I=I1∗exp(−tτrise)+I2∗exp(−tτdecay)

To determine Na^+^ apparent *K*_m_ values, nonlinear curve fitting was used with a Michaelis–Menten like equation:

(6) $$I=I_{\max}\frac{\left[{Na}^{+}\right]}{\left(K_{m}+\left[{Na}^{+}\right]\right)}$$

Here, *I*_max_ is the current at saturating substrate concentration, [Na^+^] is the concentration of Na^+^.

### MD simulations

The model system for MD simulations was generated with VMD software (55) (https://www.ks.uiuc.edu/Research/vmd/), using an ASCT2 structure (7BCS) (24). The *Lc*-BPE parameter, generated *via* the multipurpose atom-typer for CHARMM (MATCH) approach (56), using SwissParam (57). The final model was inserted in a preequilibrated POPC lipid bilayer with the dimensions of 130 × 130 × 90 Å. TIP3P water was added to generate a box measuring 100 Å in the z-direction. NaCl was added at a total concentration of 0.15 M, and the system was neutralized. The total number of atoms in the system was 136,293. Simulations were run using the CHARMM36 force field. NAMD (58) simulations were performed using 2000 steps of minimization, followed by 10 ns equilibration runs under constant pressure conditions (NPT), and then for 100 ns (59). The RMSD (calculated from the peptide backbone) increased from 1 Å soon after simulation starts to ∼3.5 Å after 20 ns of equilibration, after which it was at steady state. The cutoff for local electrostatic interactions was set to 12 Å. For long-range electrostatic interactions, we used the particle-mesh Ewald method implemented in NAMD. Bonds to hydrogen atoms and TIP3P water were kept rigid using SHAKE. The time steps of the simulations were 2 fs. The time evolution of distances and distribution function analysis were calculated by Tcl/Tk programs built in the VMD software. For distance calculations, we selected S353 (N) from the transporter and (O08) from the *Lc*-BPE as reference atoms to evaluate the distance changes of *Lc*-BPE; D475 (OD1) from the transporter and SOD 1 as reference atoms to evaluate the distance changes of Na1; D388 (OD1) from the transporter and SOD 3 as reference atoms to evaluate the distance changes of Na3.

## Data availability

All the data needed to support the conclusions of this study are presented in the article and/or supplementary materials. Raw data are available upon reasonable request.

## Supporting information

This article contains supporting information.

## Conflict of interest

The authors declare that they have no conflicts of interest with the contents of this article.
