# Supplementary material for: Transient kinetics reveal the mechanism of competitive inhibition of the neutral amino acid transporter ASCT2
Source: J Biol Chem. 2024 May 17;300(6):107382. doi: 10.1016/j.jbc.2024.107382 (PMC11193019; doi:10.1016/j.jbc.2024.107382)
Supplement: Supporting Figures S1–S8 [file mmc1.pdf]

Transient kinetics reveal the mechanism of competitive inhibition in neutral amino acid transporter ASCT2

Yang Dong, Jiali Wang and Christof Grewer

Supporting Information

List of Material Included

Figure S1. Comparison of current amplitude between $ASCT2_{WT}$ and mutants.

Figure S2. Trajectories of *Lc*-BPE/Na1/Na3 MD simulations.

Figure S3. Trajectories of *Lc*-BPE/Na1 MD simulations.

Figure S4. Trajectories of *Lc*-BPE/Na3 MD simulations.

Figure S5. Transient currents induced by application of Serine or *Lc*-BPE in the presence of 70 mM Na^+.

Figure S6. Transient currents induced by application of Serine or *Lc*-BPE in the presence of 20 mM Na^+.

Figure S7. Transient currents induced by application of Serine or *Lc*-BPE in the presence of 5 mM Na^+.

Figure S8. DL-TBOA binding is associated with biphasic ouawrd and inward charge movement in EAAC1.

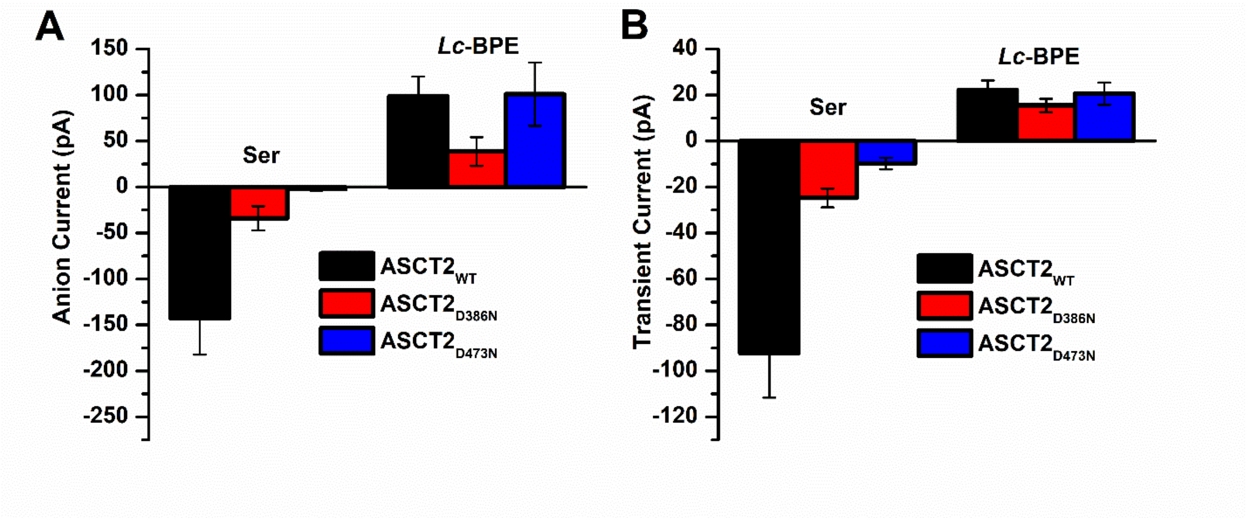

Figure S1. Comparison of current amplitude between ASCT2$_{WT}$ and mutants. A, anion currents at 2 mM serine (left panels) and 100 µM *Lc*-BPE (right panels) for ASCT2$_{WT}$ and mutants. B, transient currents at 2 mM serine (left panels) and 100 µM *Lc*-BPE (right panels) for ASCT2$_{WT}$ and mutants.

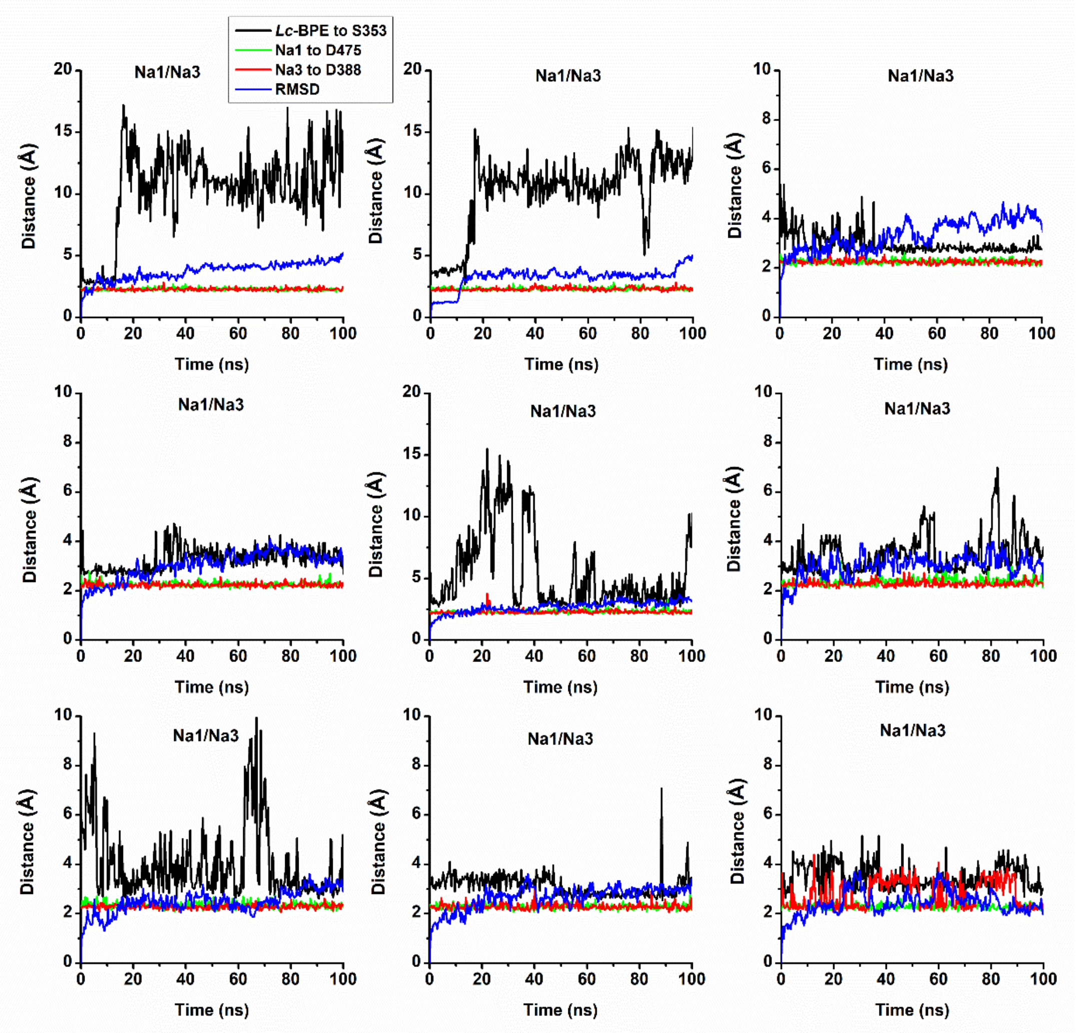

Figure S2. Trajectories of *Lc*-BPE/Na1/Na3. Distance calculation [*Lc*-BPE (black), Na1 (green), Na3 (red) and RMSD (blue)]. Distances were calculated based on atoms described in *Experimental Procedures*.

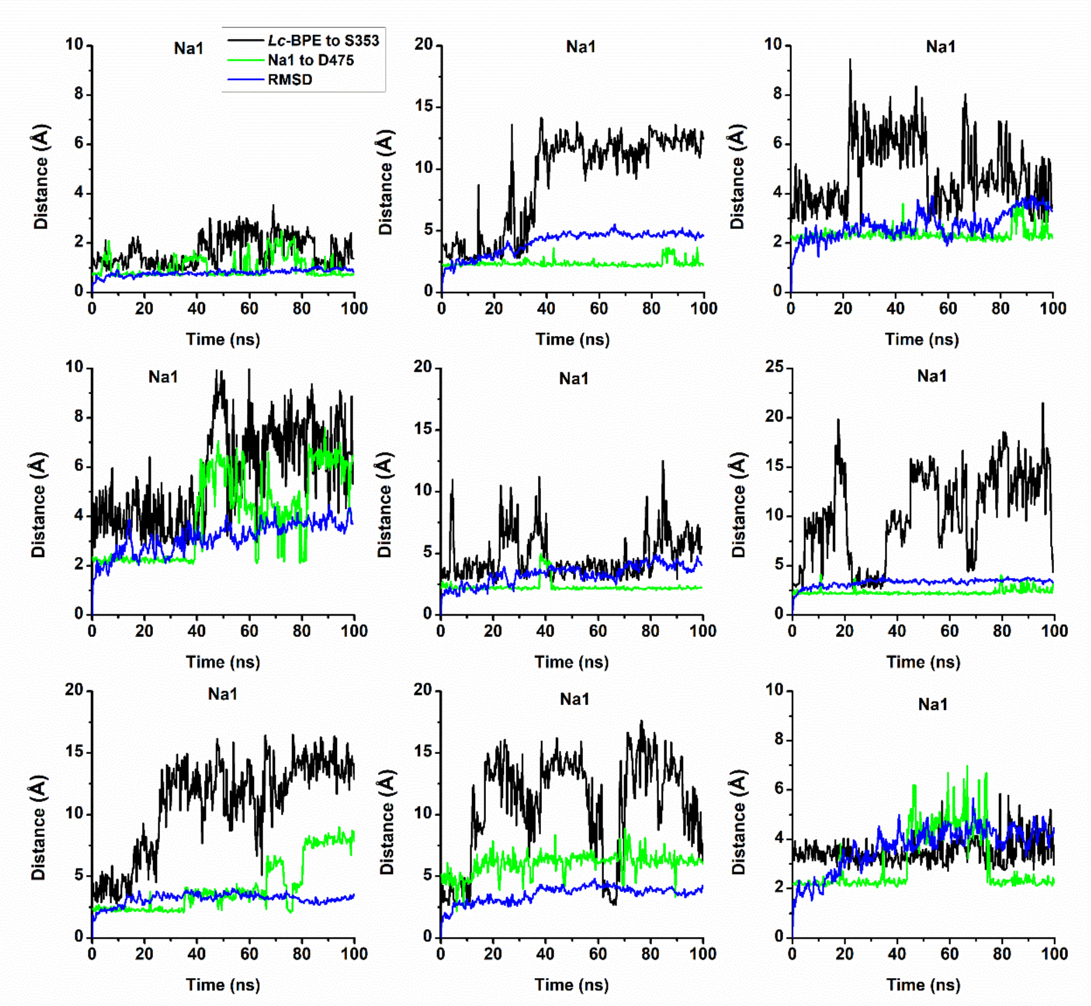

Figure S3. Trajectories of *Lc*-BPE/Na1. Distance calculation [*Lc*-BPE (black), Na1 (green) and RMSD (blue)]. Distances were calculated based on atoms described in *Experimental Procedures*.

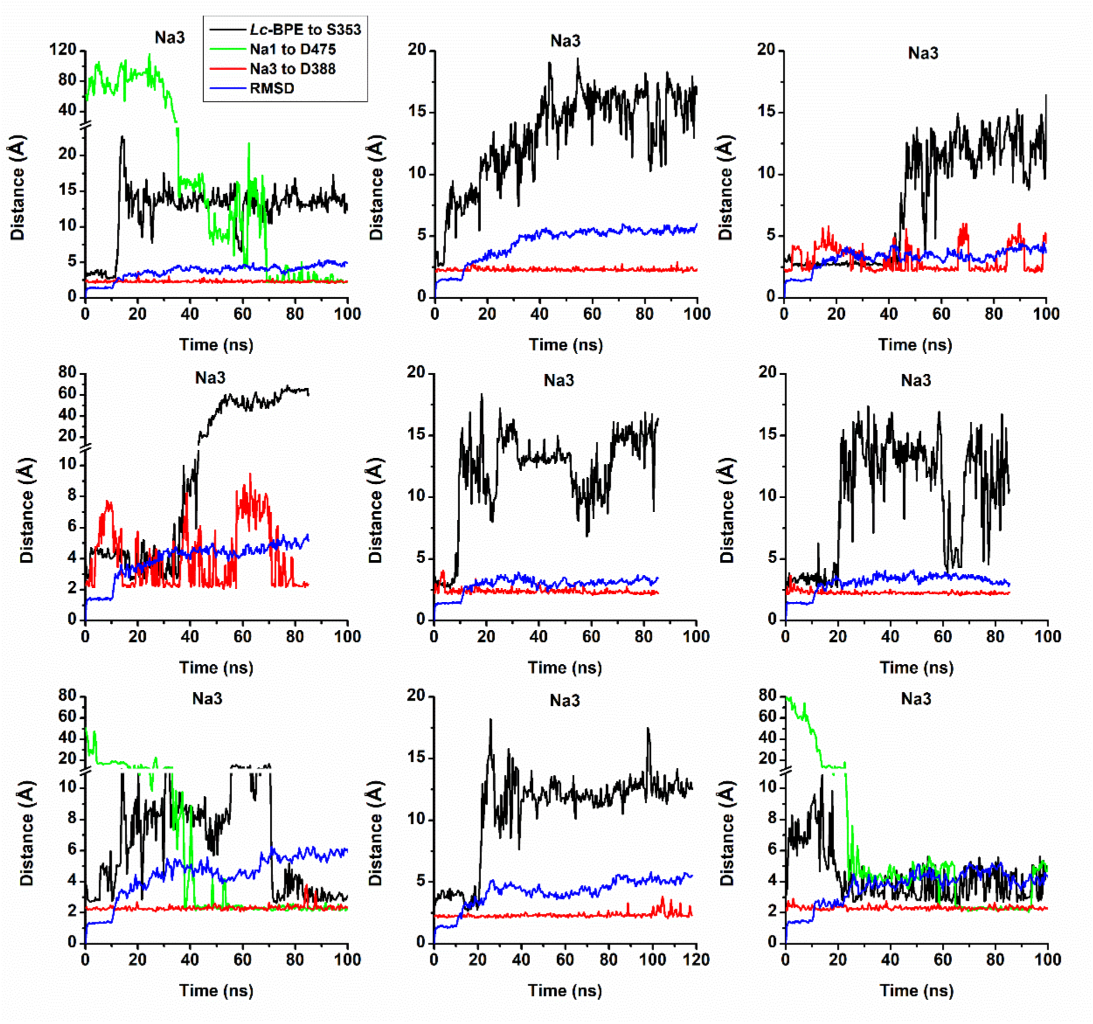

Figure S4. Trajectories of *Lc*-BPE/Na3. Distance calculation [*Lc*-BPE (black), Na1 (green), Na3 (red) and RMSD (blue)]. Distances were calculated based on atoms described in *Experimental Procedures*.

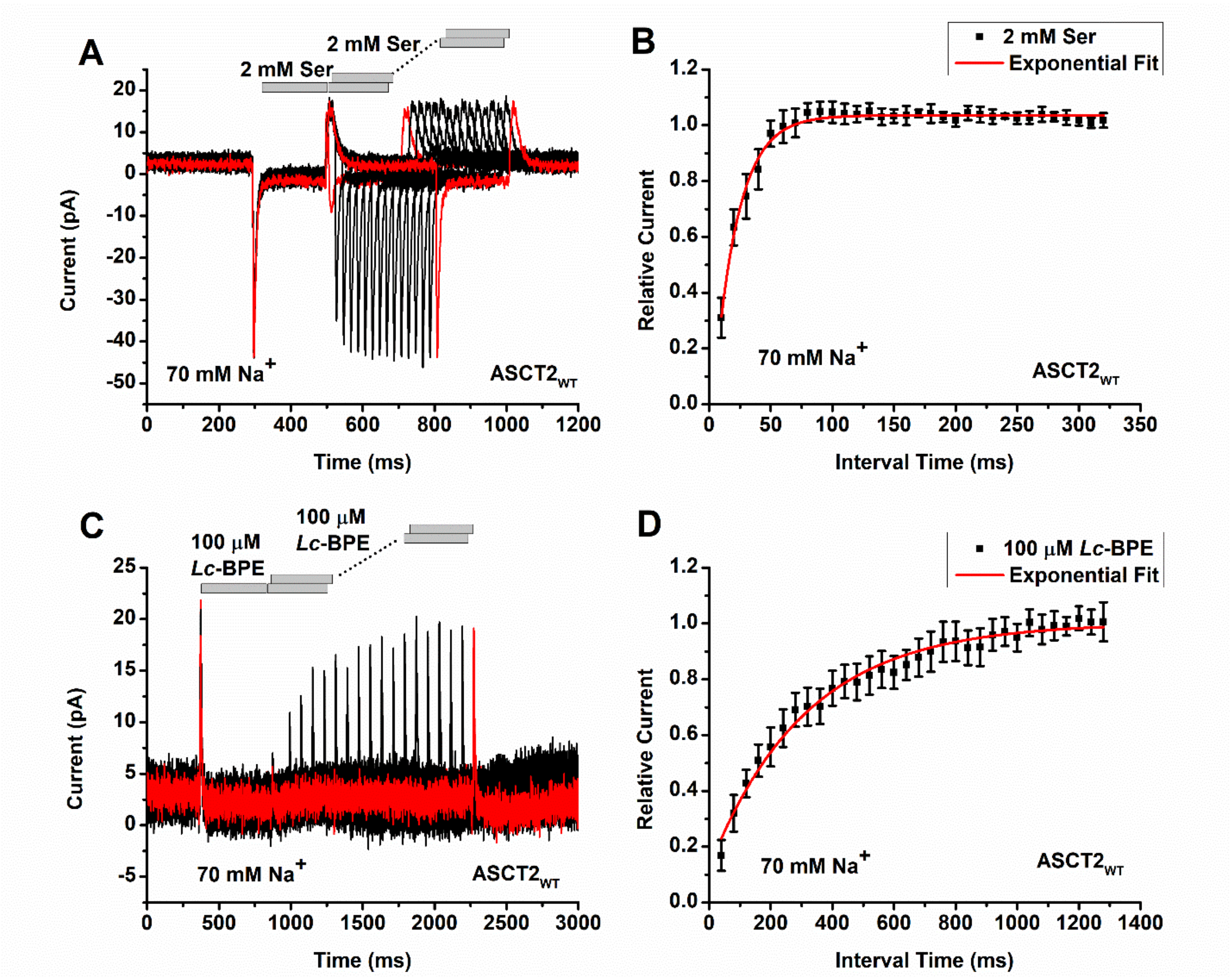

Figure S5. Transient currents induced by application of Serine or *Lc*-BPE in the presence of 70 mM Na^+. A, transient currents recorded in $ASCT2_{WT}$ in response to two consecutive pulses of 2 mM serine, or (C) 100 µM *Lc*-BPE alone application in the presence of 70 mM of Na^+, with varying inter-pulse interval (pulse protocol shown at the top, grey bars) under homo-exchange conditions. The intracellular solution contained 130 mM NaMes/10 mM serine, the extracellular solution contained 70 mM NaMes. B, recovery of the transient current in the presence of 2 mM Ser or (D) 100µM *Lc*-BPE alone. The red solid lines represent the best fits to an exponential equation with time constant of 19 ± 2 ms (serine) and 310 ± 40 ms (*Lc*-BPE alone).

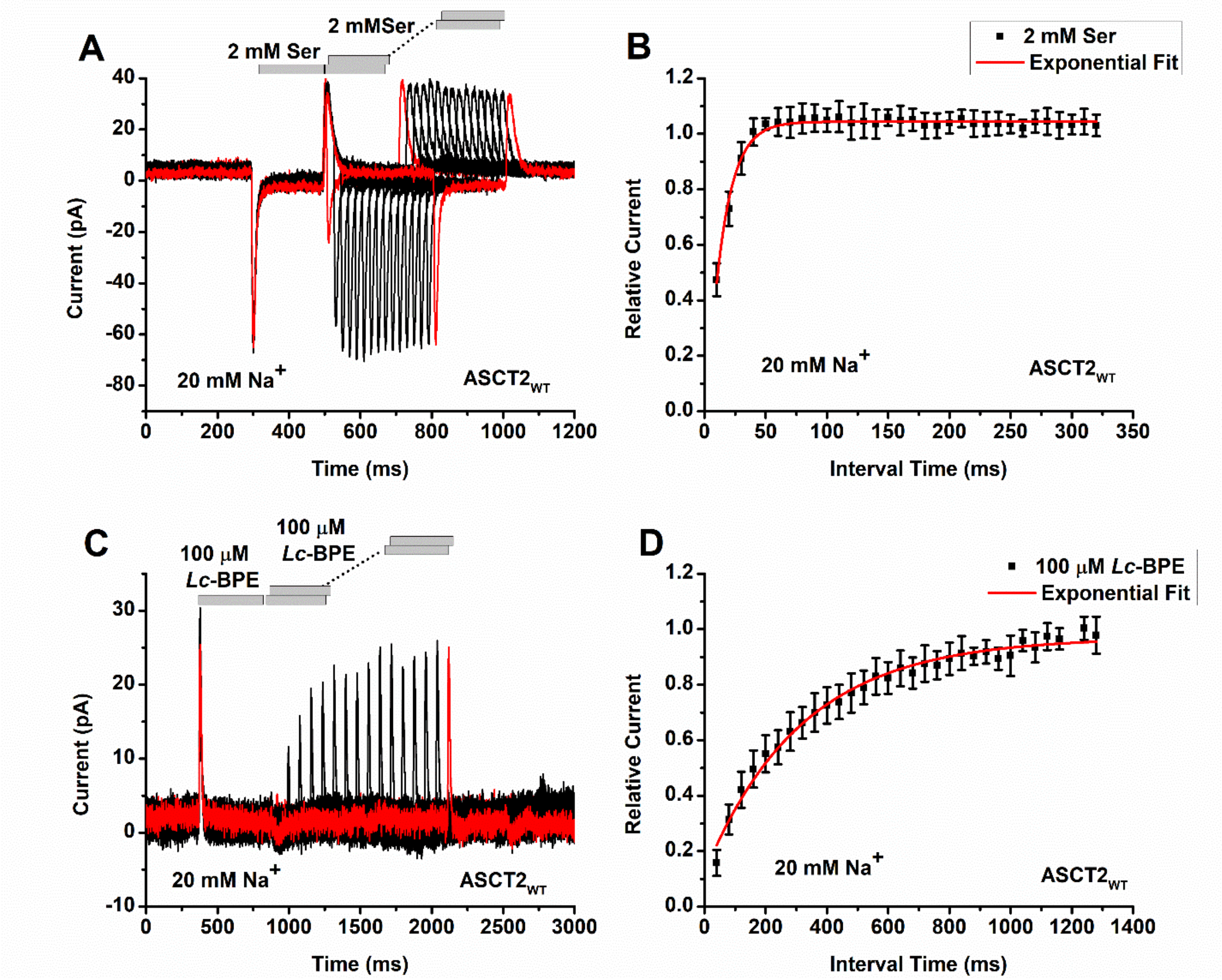

Figure S6. Transient currents induced by application of Serine or *Lc*-BPE in the presence of 20 mM Na$^+$. A, transient currents recorded in ASCT2$_{WT}$ in response to two consecutive pulses of 2 mM serine, or (C) 100 µM *Lc*-BPE alone application in the presence of 20 mM of Na$^+$, with varying inter-pulse interval (pulse protocol shown at the top, grey bars) under homo-exchange conditions. The intracellular solution contained 130 mM NaMes/10 mM serine, the extracellular solution contained 20 mM NaMes. B, recovery of the transient current in the presence of 2 mM Ser or (D) 100µM *Lc*-BPE alone. The red solid lines represent the best fits to an exponential equation with time constant of 14 ± 1 ms (serine) and 320 ± 40 ms (*Lc*-BPE alone).

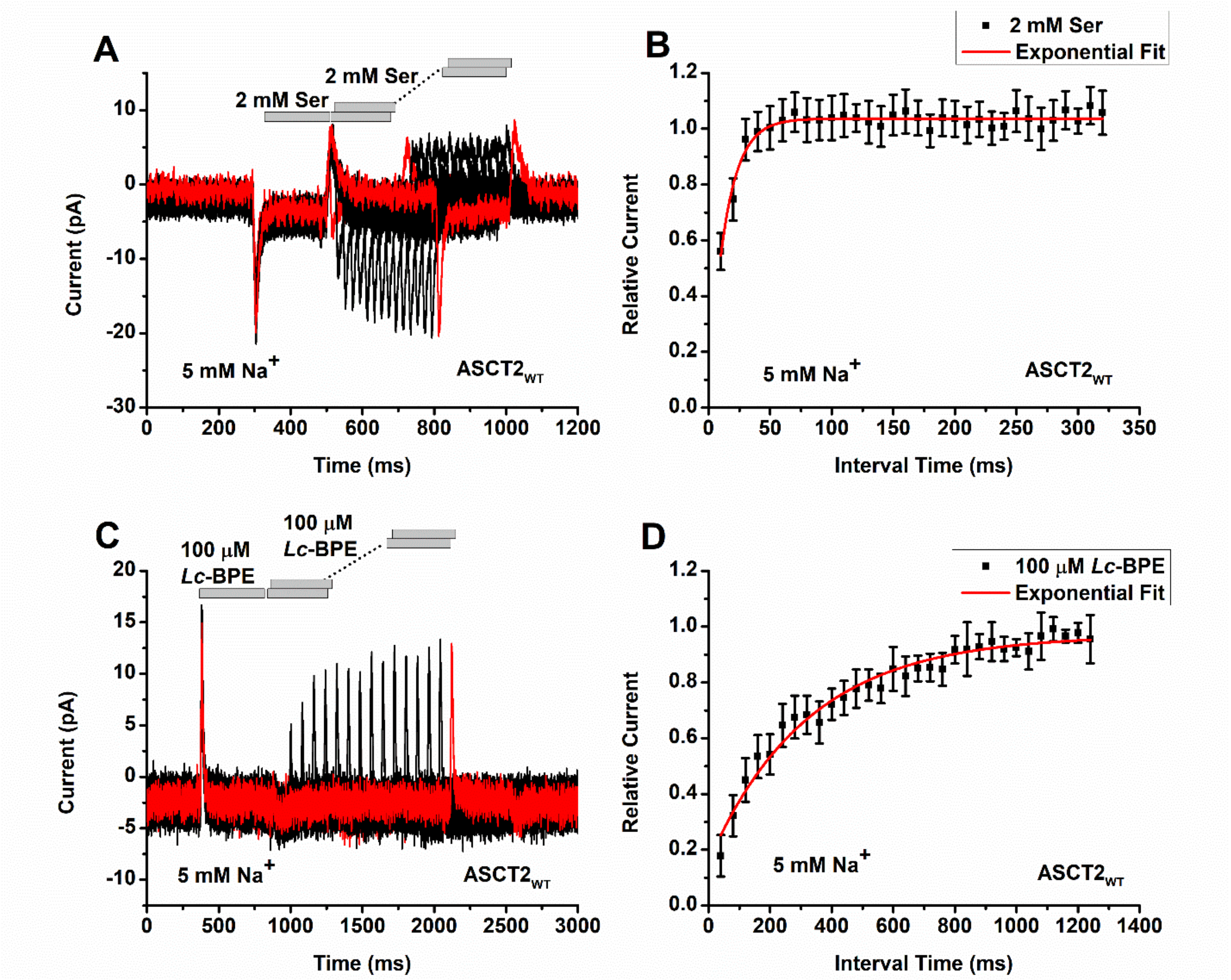

Figure S7. Transient currents induced by application of Serine or *Lc*-BPE in the presence of 5 mM Na^+. A, transient currents recorded in $ASCT2_{WT}$ in response to two consecutive pulses of 2 mM serine, or (C) 100 µM *Lc*-BPE alone application in the presence of 5 mM of Na^+, with varying inter-pulse interval (pulse protocol shown at the top, grey bars) under homo-exchange conditions. The intracellular solution contained 130 mM NaMes/10 mM serine, the extracellular solution contained 5 mM NaMes. B, recovery of the transient current in the presence of 2 mM Ser or (D) 100 µM *Lc*-BPE alone. The red solid lines represent the best fits to an exponential equation with time constant of 14 ± 3 ms (serine) and 320 ± 60 ms (*Lc*-BPE alone).

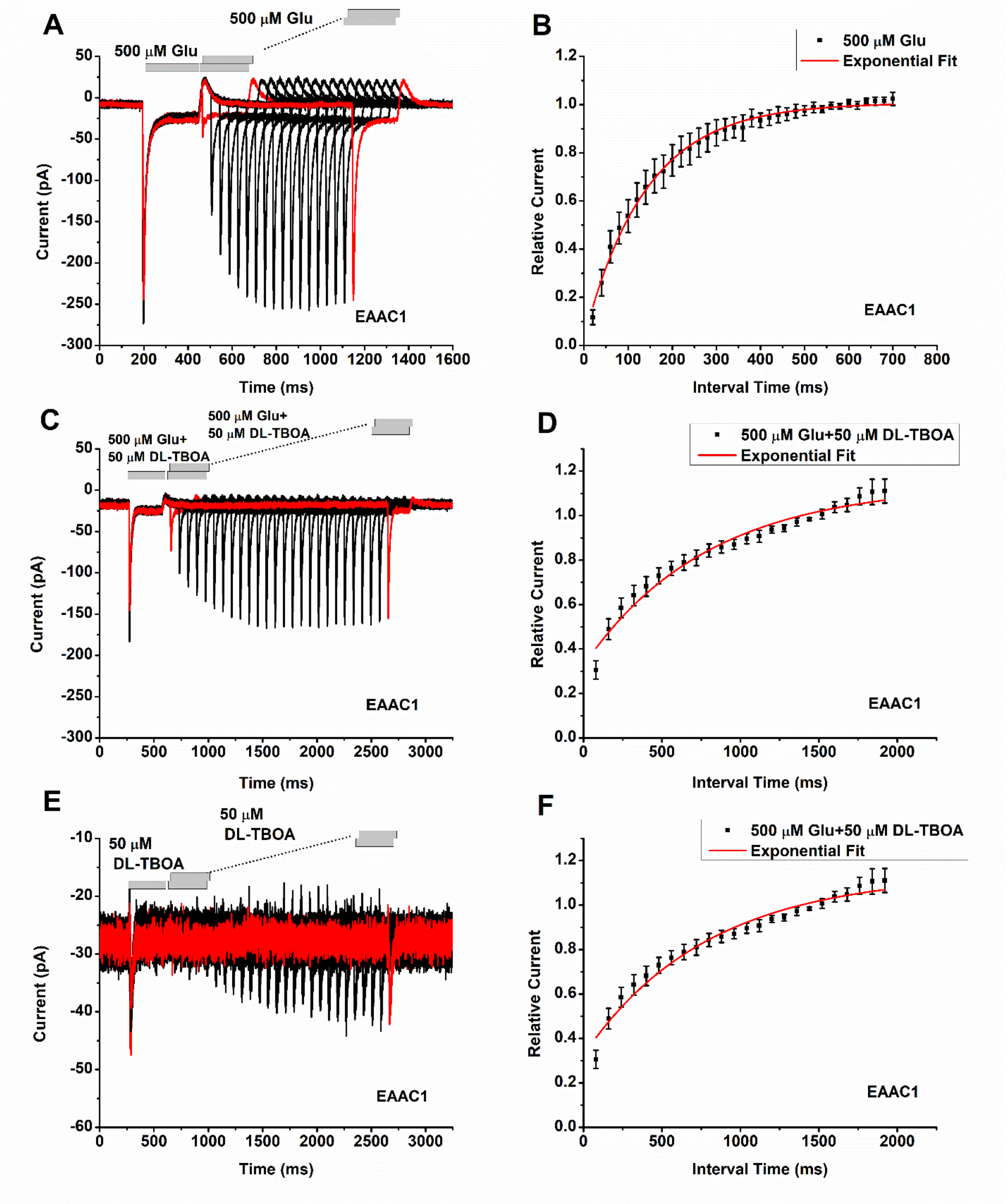

Figure S8. DL-TBOA binding is associated with biphasic ouawrd and inward charge movement in EAAC1. A, transient currents recorded in EAAC1 in response to two consecutive pulses of 500 μM glutamate, (C) 500 μM glutamate and 50 μM DL-TBOA or (E) 50 μM DL-TBOA alone application in the presence of 140 mM of Na^+, with varying inter-pulse interval (pulse protocol shown at the top, grey bars). The inset shows the time course of the transient current on

a magnified time scale.The intracellular solution contained 130 mM KMes, the extracellular solution contained 140 mM sodium NaMes. B, recovery of the transient current in the presence of 500 μM glutamate, (D) 500 μM glutamate and 50 μM DL-TBOA or (F) 50 μM DL-TBOA alone. The red solid lines represent the best fits to an exponential equation with time constant of 140 ± 9 ms (glutamate), 790 ± 200 ms (glutamate + DL-TBOA) and 961 ± 154 ms (DL-TBOA alone).
